# A bayesian network meta-analysis to explore modifying factors in randomized controlled trials: what works for whom to reduce depression in nursing home residents?

**DOI:** 10.1186/s12877-024-05117-8

**Published:** 2024-06-14

**Authors:** Ine J. N. Declercq, Ruslan Leontjevas, Peter Verboon, Patricia De Vriendt, Debby L. Gerritsen, Susan van Hooren

**Affiliations:** 1 Department of Primary and Community Care, Radboud University Medical Center, Gelderland, 6500 HBNijmegen The Netherlands; 2 Faculty of Psychology, Open University of the Netherlands, Heerlen, The Netherlands; 3 Department of Gerontology and Frailty in Ageing (FRIA) and Mental Health and Wellbeing (MENT) Research Group, Faculty of Medicine and Pharmacy, Vrije Universiteit Brussel, Brussels, Belgium

**Keywords:** Depressive symptoms, Long-term care, Nursing home, Meta-analysis, Meta-regression analyses, Moderator effects

## Abstract

**Background:**

Reviews of depression interventions in nursing home residents resulted in positive findings. However, because of the heterogeneity of the studies, it remains unclear what works for whom. Considering moderator effects may contribute to a comprehensive understanding of depression treatment in residents. Therefore, this study aims to review depression interventions, examining moderator effects of (1) residents’ factors, and (2) components specific of interventions.

**Methods:**

A Bayesian network meta-analysis of randomized controlled trials primarily aimed at reducing depressive symptoms among residents was conducted. First, intervention types, e.g., exercise interventions, were compared to care as usual. Second, meta-regression analyses were conducted for moderator effects of residents’ factors (i.e., severity of depressive symptoms, physical dependency, and cognitive impairment) and components identified as specific to an intervention (e.g., music, creativity, positivity).

**Results:**

Our search across six databases resulted in 118 eligible studies: 16 on neurobiological interventions, 102 on non-pharmacological interventions. Compared to care as usual, cognitive interventions, such as cognitive behavioral therapy and goal-oriented therapy, showed the strongest effects (MD = -1.00, 95% CrI [-1.40 to -0.66]). Furthermore, the severity of depressive symptoms moderated the effect of interventions (ƅ = -0.63, CrI 95% [-1.04 to -0.22]), while none of fifteen identified intervention-specific components did. In residents with a depression diagnosis, there were larger effect sizes for interventions including daily structure, psychoeducation, healthy food, creativity, positivity, and an activating/encouraging environment, whereas interventions focusing on distraction and relaxation had larger effect sizes in those residents without.

**Conclusions:**

By examining the moderator effects, we provided an integrative perspective on the observed variations in effects across different target groups, and components of depression interventions. This approach underscores the complex nature of interventions, emphasizing the need for continued transdisciplinary research, and the exploration of potential moderators. Future investigations should carefully assess residents’ factors and choose interventions and their components accordingly.

**Supplementary Information:**

The online version contains supplementary material available at 10.1186/s12877-024-05117-8.

## Background

Depression in nursing home (NH) residents is associated with decreased quality of life [1], increased risk of developing dementia [2], and a heightened mortality rate [3]. Approximately 30% of residents in NHs experience symptoms of depression. This is estimated three times higher than among older adults in the community [4, 5]. Therefore, it is very important to focus on prevention and early treatment of depressive symptoms (DS), which may prevent symptom worsening and could prevent the onset of a depressive syndrome [6]. Although depression interventions are effective in reducing DS in NH residents, due to heterogeneity among studies regarding methodology, intervention strategies, and participants, results of studies on reducing or preventing DS are challenging to interpret and generalize [7–9]. To better understand these inconsistencies in results, insight into potential moderator effects in interventions is needed [10].

### Interventions to reduce DS among NH residents

A wide array of interventions, can be identified to reduce depression in NH residents (see Table 1) Previous reviews show differences in intervention effects among various target groups. For example, exercise-based interventions were found to be beneficial to reduce DS in older adults and NH residents without cognitive impairment [8, 11], whereas this beneficial effect was not confirmed in another review focusing on older adults with cognitive impairment [7]. This difference in effects could stem from different needs and characteristics in specific target groups, but also from the specific components of interventions. Additionally, the delineated type of interventions exhibit substantial overlap and share common elements or components. For example, dance interventions are primarily aimed to improve aerobic capacities of residents, but share many common components with sensory stimulating interventions, such as exercise, music, and social interaction. This complexity poses an extra challenge in achieving a thorough understanding that can address the question ‘What works for whom?' Therefore, focusing on moderator effects of residents’ factors and components specific to the intervention is needed for a more comprehensive understanding. However, since previous reviews focus on a specific intervention type, or specific target group, it is challenging to align interventions with various target groups of residents. Therefore, to the best of our knowledge, this is the first review including all types of depression interventions in residents that not only considers the participant’s level of cognitive impairment but also level of physical dependency.

**Table 1:** Interventions to reduce DS

Intervention Type	Primary aim of the intervention and examples	Examples of components specific to the intervention that may contribute to the effectiveness
Cognitive Interventions	∙ Aimed to change thinking and behavioral patterns [12] ∙ Examples: cognitive behavioral therapy, goal-oriented therapy	∙ Thought reframing ∙ Social interaction
Exercise Interventions	∙ Interventions focusing on improving strength, coordination, flexibility, range of motion, endurance and aerobic capacity [13] ∙ Examples: functional training, band resistance exercise, aerobics	∙ Strength ∙ Relaxation ∙ Music ∙ Social interaction ∙ Activating
Sensory Stimulation	∙ Interventions stimulating the primary senses [14] ∙ Examples: music therapy, aromatherapy, multisensory stimulation	∙ Exercise ∙ Creativity ∙ Music listening ∙ Social interaction
Green Care	∙ Interventions including nature, natural environment and animals [15] ∙ Examples: care farming, animal-assisted therapy, horticultural therapy	∙ Small motor skills ∙ Relaxation ∙ Contact nature and animals ∙ Petting/caretaking ∙ Social interaction
Neurobiological Interventions	∙ Interventions directly targeting neurobiological mechanisms [11] ∙ SSRI, Light therapy	∙ Psychotropic drugs ∙ Sensory stimulation (light) ∙ Nutrition and food supplements
Tailored Interventions	∙ The least restrictive and least costly intervention adapted to the specific needs of individual residents [16] ∙ Examples: shared care, staff education, psychoeducation, tailored care planning	∙ Psychoeducation ∙ Activating/encouraging ∙ Learning ∙ Social interaction

A complete overview of this summary can be found in Additional File 1. Classification of interventions in the included studies was based on the key component of interventions, as presented in Table 1 and described in the respective reference

### Moderator effects of residents’ factors

Losses in abilities (e.g., being physical dependent and/or having cognitive impairment) are viewed as predictors for NH admission and risk factors for developing DS [17, 18].Therefore, these risk factors could be seen as potential moderators that may impact the effectiveness of depression interventions in residents. For instance, among NH residents without cognitive impairment, physical exercise was found to be one of the most effective intervention in reducing DS [8], whereas the beneficial effects of exercise were not confirmed in another review focusing on older adults with cognitive impairments [7].

As a second example, reduced untargeted stimuli due to COVID-19 restriction policies in NHs seemed to be beneficial for reducing DS among residents with cognitive impairment, whereas residents without cognitive impairment were negatively affected [19].

Both examples suggests that different effects may occur in specific target groups, and underscores the importance of more insight into the moderator effects of various residents’ factors. These differences in intervention effectiveness among the different target groups of residents may stem from different needs and characteristics in these target groups [20]. However, to the best of our knowledge, no reviews have been published focusing on participants’ physical status.

### Moderator effects of components specific to the intervention

Alongside residents’ factors, components specific to the interventions can be considered as potential moderators. These components are specific parts of the intervention that are expected to contribute to the reduction of DS (see Table 1). The social component is an example of previously investigated intervention-specific component. Although several reviews reported that group-based activities were, in the long term, not more effective in reducing DS than individual interventions [21, 22], researchers highlighted the importance of the social component [23, 24]. For example, feelings of being socially connected [24] and the enjoyability of the interaction in group activities [23] could impact the effectiveness of the group-based interventions. Another example concerns the use of music in activities. Active music interventions are often combined with dancing or other physical activities such as playing the instruments. Because music has the potential to improve physical performance and physiological efficiency [25], the use of music in physical activities may contribute to better improvements in balance, walking, and functional abilities in NH residents, and subsequently result in reduced DS [26, 27]. However, other researchers found that receptive music interventions are more likely than active music interventions to be effective in reducing DS in NH residents [28]. Listening to music may evoke autobiographical memories mainly accompanied by strong positive emotions. This may be rewarding, may reduce level of stress, and may enhance feelings of self-esteem [29, 30]. Moreover, the effect of these components might differ across the various target groups. It is possible that people with DS, who tend to overgeneralize negative memories and have difficulties in retrieving positive memories [31], are more likely to benefit from receptive music interventions, whereas active music interventions may be more beneficial for residents with reduced physical abilities as these may improve their functional abilities. More insight is needed into how different intervention components, such as social interaction and the way music is incorporated in the intervention, contribute to the effects of the interventions.

### Objective and rationale of the current review

To summarize, the current state of the literature is inconclusive about the effects of interventions in reducing DS in nursing home residents, which limits researchers to make strong statements and recommendations for practitioners to apply interventions as effectively as possible.

Our first objective was to gain more insight into the relative effectiveness of interventions in reducing DS in NH residents, based on the available best evidence provided in randomized controlled trials (RCT)[32]. Our second objective was to gain insight into moderator effects of residents’ factors. This review explored risk factors for DS in NH residents, which included severity of DS, physical dependency, and cognitive impairment. The third objective of this meta-analysis was to explore how the effectiveness of interventions may be modified by the different components specific to the interventions.

## Methods

We performed a Bayesian Network Meta-Analysis (NMA) to gain insight into the relative effectiveness of interventions in reducing DS for NH residents and factors that may influence this effectiveness.

### Eligibility criteria

RCTs were included based on the following PICOS criteria: (1) Participants residing in long-term care facilities, (2) Interventions aiming to prevent or reduce DS, (3) Comparing an experimental group to a control group, waiting-list, placebo-controlled and/or another experimental group receiving a different intervention, (4) primary Outcome focused on DS, assessed with a standardized measurement tool, (5) and Study design involved original research articles that randomly assigned participants, or were described as randomized controlled trials as these are seen as the “gold standard” for effectiveness research and randomization reduces bias [33]. Subsequently, studies not meeting these criteria, such as noninstitutionalized participants, studies where depression was not a primary outcome, single-armed studies, were excluded. See Additional File 2, Table 2.1 for a detailed overview of these criteria.

### Information sources and search strategy

We selected CENTRAL, PubMed (MEDLINE), and EMBASE as core databases [34]. Additionally, subject-specific databases included APA PsycInfo (databases for behavioral and social sciences) and Cumulative Index to Nursing & Allied Health Literature (CINAHL). Finally, we searched Web Of Science (a citation database). All databases underwent two searches: the first in August 2021 and the second in July 2022. In addition, we inspected the reference sections of reviews on the effects of interventions on DS among NH residents [7, 8, 11, 35–37] to identify qualifying studies. The search string was developed to gain a comprehensive perspective, meaning that we did not exclude any type of intervention or specific target group of residents, such as participants with or without certain cognitive impairment, or age categories. We consulted MeshTree (PubMed) and Emtree (Embase) to determine relevant search terms for criteria representing participants and outcome. In addition, we used predefined search strings for RCT studies [34, 38, 39]. Although no filters were set on publication date and language, we only included articles written in English, Dutch, German, French, and Spanish due to limited knowledge of other languages. The complete search strategy is available in Additional File 2, Table 2.2.

### Study selection and data collection

After using EndNote (version 20.3) to remove duplicates, Rayyan software [40] was employed to screen and select for eligibility. For each study, two reviewers (ID, the first author, and four interns, namely, IC, MA, LS, and MB) independently assessed studies based on title and abstract. This process occurred step by step, with differences between the reviewers being regularly discussed. Selection based on full text, further data extraction, and quality assessment were done in Microsoft PowerApps, Word, and Excel using a decision tree and a self-developed protocol (See Additional File 2, Fig. 2.1 and Protocol for Data Extraction). This protocol was based on the Consolidated Standards of Reporting Trials (CONSORT Guidelines), the quality assessment tool for quantitative studies resulting from the Effective Public Health Practice Project (EPHPP), and contained additional questions regarding the moderators of interest [41, 42]. Based on a review on neuropsychiatric symptoms of dementia in NH [9], we categorized studies by intervention type using the following designations: sensory stimulation, exercise interventions, cognitive interventions, neurobiological interventions and tailored interventions. After article selection was complete, we expanded this typology by adding psychosocial interventions, green care, pet-robots, and reminiscence, since these were lacking. In addition, we added the following control groups: care-as-usual (CAU), waiting-list, placebo interventions, and neurobiological placebos. Classification of interventions was based on the key component of interventions (See Table 1). Regarding moderator effects of residents’ factors, we focused on risk factors for developing DS in residents [17, 18], namely severity of DS, physical dependency, and cognitive impairment. For each of these risk factor, studies were assigned one of the following labels: “Yes” (indicating the risk factor was present), “No” (indicating the risk factor was not present), or “Can’t tell” when nothing was mentioned. Criteria for labelling these moderators were based on descriptive information in the article texts (e.g., “participants had to be diagnosed with dementia to be included in the study;” “all participants used a wheelchair or were not able to walk without assistance”). Mean baseline scores on standardized measurement tools were used only if information regarding residents’ factors was missing (See Additional File 2, Fig. 2.1). This strategy of step-by-step labeling studies was chosen to limit potential heterogeneity among participants in the different subgroups, and to minimize data aggregation. For labeling moderating components specific to the interventions (See Additional File 2, Fig. 2.1), categories were based on a group concept mapping procedure, which aimed to determine actions to improve mood in residents, from the perspective of healthcare workers and residents themselves [19].

The first author (ID) completed the further study selection and data extraction process, which was then repeated by a second reviewer (six interns, namely, IC, MA, LS, MB, SK and LS). Disagreements were first resolved between the two reviewers and, if necessary, further discussed with the second author (RL). If required, missing information or ambiguities were checked with the corresponding authors. To limit potential subjectivity, a third reviewer checked components specific to the interventions in almost half (43%) of all included studies to ensure a consistent interpretation of these elements. These reviewers are experts in geriatric care research (RL, DG, PDV), and creative arts therapies (SVH).

### Bias and certainty assessment

The EPHPP tool [42] was used to assess potential study limitations, and Eggers’ Test to detect publication bias [43]. Quality of evidence was rated using the Grading of Recommendations Assessment, Development and Evaluation (GRADE) principles for NMA [44–46]. The GRADE-tool provides guidance to evaluate the quality of evidence resulting from our quantitative analyses and is described in detail in Additional File 3 [44–46]. The first author (ID) rated bias and certainty assessment. A second reviewer (six interns, namely, IC, MA, LS, MB, SK and LS) repeated assessments regarding study limitations for all studies, and the second author (RL) repeated assessments for almost half of all included articles (See Additional File 3, Quality Assessment).

### Quantitative Analyses

The packages *dmetar, gemtc, and rjags* for R, version 2022.2.3.492 [47], were used for the quantitative analysis [48]. Effect sizes (Hedges’ *g*) were calculated using post-test mean values (M), standard deviations (SD), and number of participants (N) in the experimental (*n*_*1*_) and control (*n*_*2*_) condition. Values were initially processed following the guidelines presented in the Cochrane Handbook [49], or, if this was not feasible, requested from the authors. If it was not possible to obtain Hedges’ *g*, studies were excluded from the review. For all studies, the standard error in the reference group was specified and, if necessary, imputed for three-armed trials [50].

The Markov Chain Monte Carlo (MCMC) sampling, with non-informative prior distribution, was used to estimate the posterior distribution. To assess convergence of the model and choose the best model fit, the multivariate Potential Scale Reduction Factor (PSRF) was calculated, with the simulated model reporting a value closer to 1 being the most preferrable [48, 51]. The constructed Bayesian network comprises both direct and indirect comparisons between the reported interventions. Direct evidence represents the results from studies where treatments were compared directly and reported in articles. Indirect evidence was obtained by estimating the effects for treatments that were not directly compared within the included studies, and was deduced on the available direct evidence [52]. The node-split method was used to check inconsistency between direct and indirect evidence [53] and to identify outliers by screening the forest plot and Bayesian p-value [54]. Outliers were excluded for quantitative syntheses. Additionally, mean difference (MD) of each intervention type was compared to CAU.

Further, network meta-regressions (NMRs) were conducted to explore the moderator effects of residents’ factors and components specific to the intervention. These moderator effects were priori selected based on previous research [17–19] and predefined in the protocol to enhance uniformity among reviewers (See Additional File 2, Protocol for data extraction). In addition, NMRs were used to detect moderator effects of the EPHPP score. A difference in deviance information criterion (DIC) of 10 was considered important to detect moderators [55]. Post-hoc analysis included additional NMAs, sensitivity analyses, and NMRs conducted in separate subgroups. Results of the NMAs and NMRs are presented in tables and forest plots.

### Transparency and openness

The study was preregistered at PROSPERO (ID CRD42021276732). The preferred reporting items for systematic reviews and meta-analyses (PRISMA) were used to report the results [56]. The datasets used and analysed during the current study are available from the corresponding author on reasonable request.

## Results

### Study Selection

**Fig. 1:**
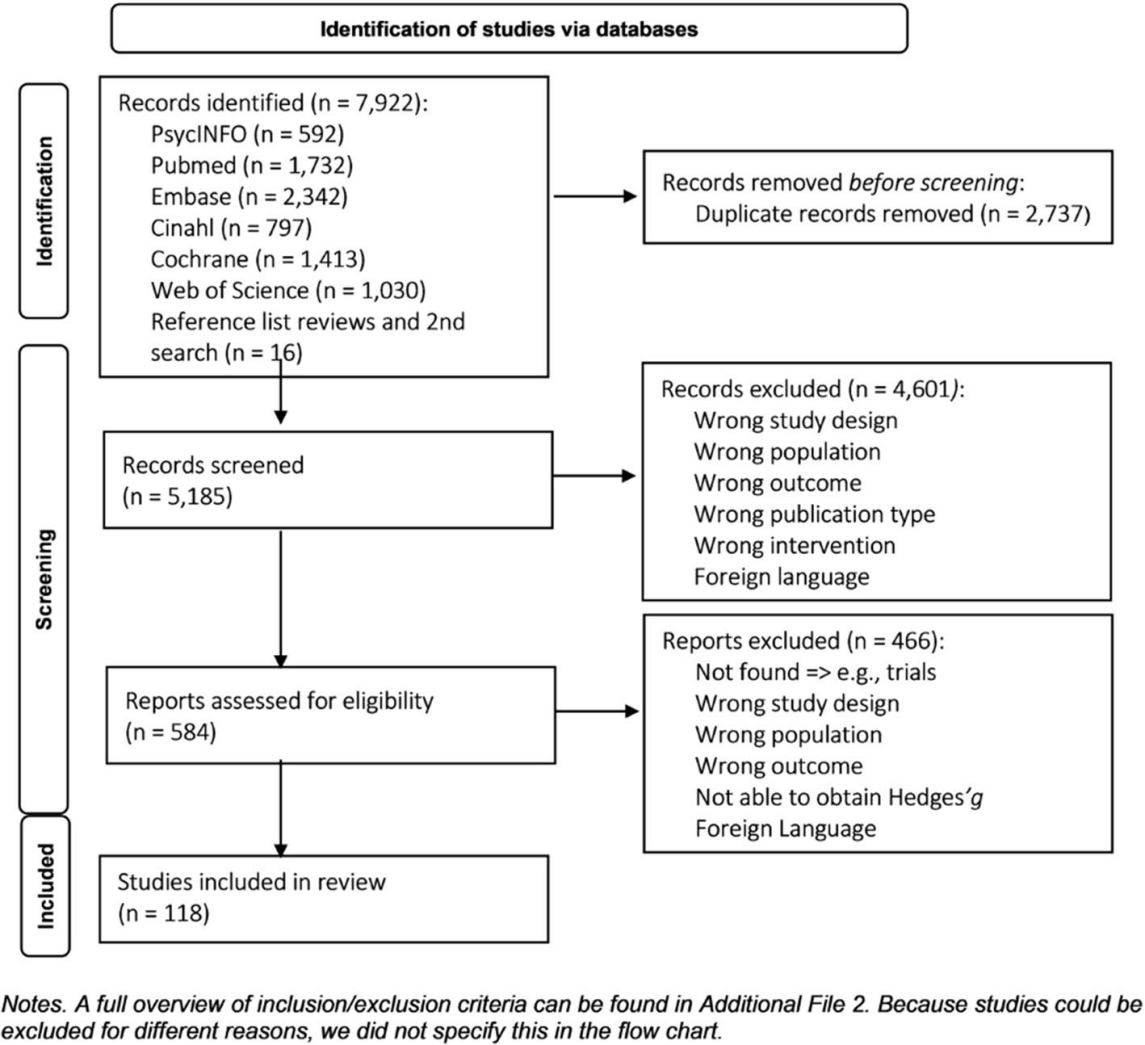
PRISMA Flow Chart [56]

Our search strategy resulted in 5,185 unique studies which were further screened based on title and abstract. After assessing full text of 584 reports, 118 studies were eligible (Fig. 1).

### Study characteristics

Most studies (78 of 118, 66%, see Additional File 4 for an overview) were conducted after 2011, with an increasing number of studies involving sensory stimulation (18 studies compared to one before 2012), green care (eight studies compared to three before 2012), and pet-robots (four studies compared to zero before 2012). Trial duration ranged from 1 to 60 weeks, with 8 weeks (18 studies, 15%), 12 weeks (26 studies, 22%), and 24 weeks (14 studies, 12%) being the most reported trial durations. Self-rated depression scales were used more frequently (86 studies, 73%) than proxy-rated scales (32 studies, 27%), especially in studies including participants with depression diagnosis. When studies involving participants with depression diagnosis are compared to studies without depression diagnosis, the frequency of use for the self-rated depression scale is, respectively, 81% to 62%.

Of the 118 studies, 65 (55%) showed a statistically significant reduction of DS compared to the control group. Most of these interventions were beneficial compared to a placebo control group (five studies), CAU (44 studies), waiting-list (five studies), another intervention within the same intervention type (six studies; e.g., an active versus a passive music therapy). Five studies were statistically beneficial when compared to another type of intervention. Participants’ physical status was reported in 43 studies, 32 of which (27%) included participants who were more physically dependent, and 11 of which (9%) included participants who were physically independent. Participants were physically dependent in more than 30% of the studies on exercise, neurobiological, psychosocial, and green care interventions. Of the studies reporting participant cognitive status, 69 studies (58%) included participants with (mild) cognitive impairment, and 28 studies (24%) concerned participants without cognitive impairment. In neurobiological interventions, sensory stimulation, and interventions with pet-robots, more than 65% of the studies concerned participants with cognitive impairment. In the other intervention types, cognitive status of participants was more equally divided among the different studies.

The interventions combined a maximum of nine different components (median = 4). Overall, green care interventions used the highest variety of components specific to the intervention with minimally two and maximally nine components (median = 5). Of the 11 green care interventions, seven focused on improving small motor skills, eight on providing warmth and comfort, five on relaxation through caressing or hugging, and four used thought-stimulating components. These thought-stimulating activities and other mind-related components, such as memories and learning activities, were most often used in cognitive interventions. In addition to those mind-related components, sensory stimulating interventions and psychosocial interventions were often combined with creativity, exercise, and music. Except for neurobiological interventions, interventions involved contact with others most of the time (93 studies, i.e., 79% of the reported studies). Of those 93 studies, 42 (36%) concerned performing activities in groups, and 38 (41%) interventions focused on sharing emotions, memories, and thoughts with peers (32 studies) or others (family or volunteer, six studies). The remaining 13 studies concerned individual contact with a therapist.

### Construction of the network

After removing two outliers based on screening the forest plot [57, 58], no significant Bayesian p-values among the different comparison arms were found, suggesting consistency between direct and indirect evidence. In addition, five single-armed studies were excluded [59–63]. The remaining 111 studies that were kept in the analysis represented 8,906 participants and 13 different types of interventions. Most studies (104 studies) were two-armed; the other seven studies were three-armed. The network was connected and consisted of 33 comparisons, with the most direct evidence for CAU compared to reminiscence (*N* = 12 comparisons), sensory stimulation (*N* = 10), cognitive interventions (*N* = 10), and psychosocial (*N* = 10) interventions. The neurobiological interventions were most often placebo controlled (*N* = 10).

### Risk of Bias and certainty of evidence

**Fig. 2:**
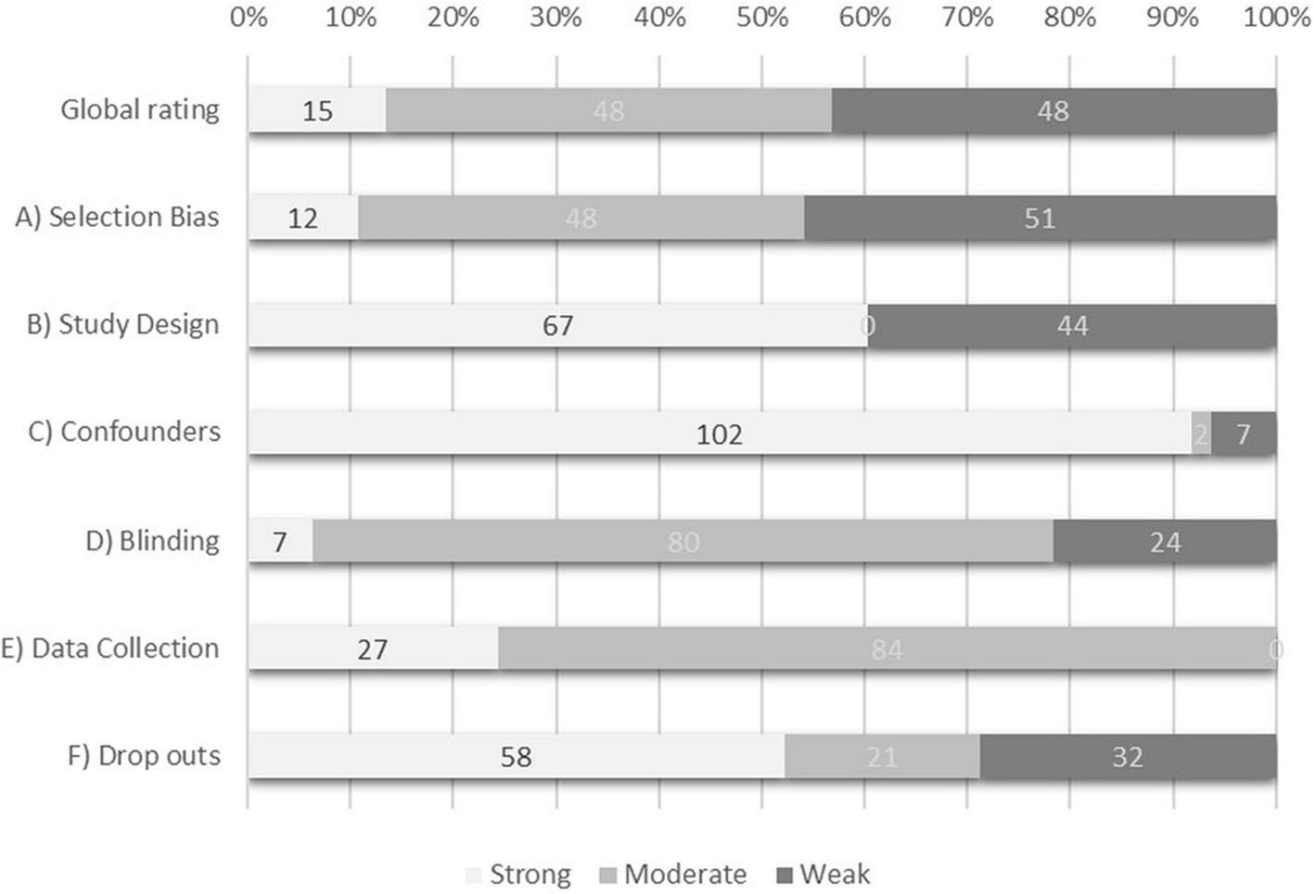
Study Limitations Assessed With the EPHPP Tool

Overall, for each GRADE assessment, quality of evidence was rated moderate. Limitations of studies mostly regarded selection procedures, study design, blinding procedures, and poor description of completers. With respect to selecting participants, 48 studies were rated as moderate, because only 60–79% of the randomly selected residents provided consent (all were invited to participate, or selection took place before consenting) or the number of residents who did provide consent was not mentioned. In 11 other studies that randomly selected participants, less than 60% of those selected agreed to participate. Those studies were therefore rated as weak, together with 40 studies that did not describe their selection procedure. Although random allocation was conducted and reported in most studies, 44 studies did not report their method of randomization and were therefore rated as weak on study design. In 12 studies, participants were blinded to the intervention. Seven of those 12 studies also had blinded assessors. In total, 32 studies were rated as weak on the category dropouts. In 13 of these, less than 60% of the participants completed the study. The other 19 studies did not describe dropouts, or dropouts could not be derived from figures, graphs, or tables. Results of the EPHPP assessment are summarized in Fig. 2, and a detailed overview can be found in Additional File 3, Table 3.1. The EPHPP assessments did not significantly moderate the NMA results (Table 2).

**Table 2:** Network Meta-Regression Analyses for the EPHPP Assessments

	N	ƅ(SD)	95% CrI	DIC
NMA				222.35
Global Rating	48	-0.31 (0.20)	-0.71 to 0.09	191.62^b^
A rate	51	-0.13 (0.20)	-0.53 to 0.27	216.79
B rate	44	-0.08 (0.21)	-0.50 to 0.34	220.65
C rate	7	-0.71 (0.37)	-1.45 to 0.01	196.22^b^
D rate	24	-0.03 (0.25)	-0.52 to 0.48	222.41
E Rate^a^	84	-0.12 (0.23)	-0.57 to 0.33	219.06
F rate	32	-0.18 (0.24)	-0.64 to 0.29	215.76

All variables are dummy-coded; *Reference Category *moderate – no bias; *ƅ* estimated regressor; *SD* Standard Deviation, *95% CrI* 95% Credible Interval, *DIC* Deviance Information Criterion

^a^Reference Category no bias

^b^Differences in DIC of more than 10 compared to model with N included studies

Inconsistency between direct and indirect evidence, i.e., substantial difference between direct and indirect evidence, was rated as moderate for most of the comparison arms (see Additional File 3, Table 3.2). Heterogeneity varied greatly with I2 ranging from zero for psychosocial interventions to pet-robots and exercise interventions, and for pet-robots compared to CAU, to 96% for cognitive interventions compared to CAU. The mean sample sizes of the included studies, together with the number of included studies per comparison arm, was rather low to draw a strong conclusion. Therefore, imprecision of the NMA was also rated as moderate. With respect to indirectness of the NMA, most of the comparison arms were rated as moderate. We used strict inclusion criteria, which made the population and interventions applicable to answer the intended research questions. Finally, analysis of the funnel plot (see Additional File 3, Fig. 3.1) and an additional check using Eggers’ test (Eggers’ test = -2.95, *p* = 0.07, *N* = 118) indicated that there was no asymmetry. However, repeating Eggers’ test, only including studies in the analyses, indicated the presence of funnel plot asymmetry (Eggers’ test = -2.11, *p* = 0.01, *N* = 111; see Additional File 3, Fig. 3.2). Therefore, publication bias seems likely, and was rated as moderate.

### Relative effectiveness of interventions (NMA)

Compared to CAU, cognitive and exercise interventions were the most effective (MD = -1.00, 95% CrI [-1.40 to -0.66], and MD = -0.97, 95% CrI [-1.30 to -0.60], respectively; Table 3). SUCRA values ranged from 8% for the placebo interventions to 90% for cognitive interventions, with a median value of 43% for tailored interventions.

**Table 3:** Network Meta-Regression Analyses for Resident Factors

	***N***	***ƅ(SD)***	***95% CrI***	***DIC***
Depression	73/127	-0.62 (0.21)	-1.03 to -0.21	94.44^a^
Physical Dependency	35/47	0.16 (0.46)	-0.75 to 1.07	82.84
Cognitive Impairment	73/104	-0.21 (0.24)	-0.68 to 0.26	173.71^a^

All variables are dummy-coded

*N* number of labeled studies per covariate/number of studies in analysis, *ƅ* estimated regression coefficient with negative values indicating better treatment outcomes on severity of DS, *SD* Standard Deviation, *95% CrI* 95% Credible Interval, *DIC* Deviance Information Criterion

^a^Differences in DIC of more than 10 compared to model with N included studies

### Moderator effect of residents’ factors (NMR)

NMRs showed that severity of DS moderated the effect (*ƅ* = -0.63, CrI 95% [-1.04 to -0.22]), suggesting that studies including participants with larger severity of DS reported stronger effects compared to CAU. Level of physical dependency and cognitive impairment did not moderate the effect (*ƅ* = 0.16, CrI 95% [-0.75 to 1.07], and *ƅ* = -0.21, CrI 95% [-0.68 to 0.26] respectively; Table 4.). Additional sensitivity analyses that considered studies with missing information about level of physical dependency and/or cognitive impairment as a middle category did not show results to conclude differently regarding moderation effects.

**Table 4.**
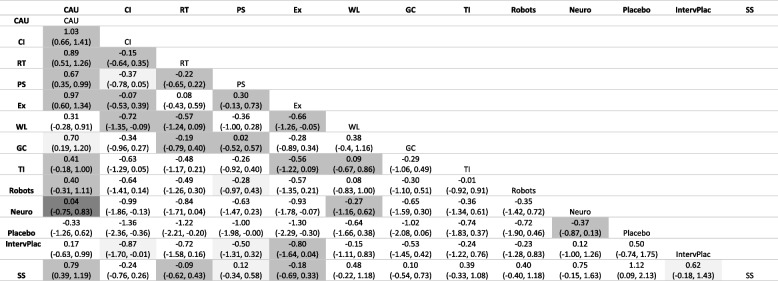
Relative Effectiveness of Interventions Based on Direct and Indirect Evidence (N=118 studies)

Mean Differences (95% Credible Interval) NMA was based on 5,000 burn-in iterations, and 100,000 actual simulation iterations, multivariate PSRF=1.0008, *Light Grey *inconsistency rated “good”, *Grey* inconsistency rated “moderate”; *Dark Grey* inconsistency rated “weak”

*CAU *Care as Usual, *CI* Cognitive Interventions, *RT *Reminiscence Therapy, *PS *Psychosocial Interventions, *Ex *Exercise Interventions, *WL *Waiting-List, *GC *Green Care, *TI *Tailored Interventions, *Robots *Pet-Robots, *Neuro *Neurobiological Interventions, *Placebo *Placebo Neurobiological Interventions, *IntervPlac *Placebo Intervention, *SS *Sensory Stimulation

### Moderator effects of components specific to the intervention (NMR)

Since severity of DS moderated the effect, we explored the components specific to the interventions separately for studies with “no depression diagnosis” (i.e., without diagnosis or mean cut-off scores under threshold value) and studies with “depression diagnosis” (i.e., minor, moderate, and major depression). Results of these additional NMRs are presented in Table 5. Although no moderator effects of these components specific to the intervention were found in both groups, the NMRs suggest that interventions with exercise (*ƅ* = -0.46, CrI 95% [-1.11 to 0.19]), relaxation (*ƅ* = -0.65, CrI 95% [-1.28 to 0.01), and nature (*ƅ* = -0.35, CrI 95% [-1.37 to 0.66]) had better effects in the group with no depression diagnosis, compared to the CAU (see Table 5). With respect to studies with participants with a depression diagnosis (see Table 5), interventions including health-related components (*ƅ* = -0.85, CrI 95% [-1.75 to 0.06]), creativity (*ƅ* = -0.54, CrI 95% [-1.64 to 0.54), contact with others (*ƅ* = -0.66, CrI 95% [-1.68 to 0.37]), and positivity-related components (*ƅ* = -0.40, CrI 95% [-1.02 to 0.22]) resulted in larger effect sizes compared to the CAU.

**Table 5:** Network Meta-Regression Analyses for Components Specific to the Intervention

	***Examples***	Participants With Depression Diagnosis (N = 73)				Participants Without Depression Diagnosis (N = 54)			
		***N***	***ƅ(SD)***	***95% CrI***	***DIC***	***N***	***ƅ(SD)***	***95% CrI***	***DIC***
NMA					122.85				101.29
Exercise	Small motor skills, coordination, ROM, strength, balance, aerobic, dance, ADL-training, yoga, tai chi	19	0.39 (0.37)	-0.35 to 1.12	103.99^*b*^	21	-0.46 (0.33)	-1.11 to 0.19	61.50^*b*^
Touch	Massage, touching animals	11	-0.18 (0.45)	-1.06 to 0.71	121.68	10	-0.09 (0.47)	-1.01 to 0.82	100.83
Relaxation	Mindfulness, breathing exercises, yoga, tai chi, massage	10	-0.01 (0.54)	-1.07 to 1.07	123.00	13	-0.65 (0.33)	-1.28 to 0.01	51.44^*b*^
Health	Health education, structure, supplements, and healthy food	12	-0.85 (0.46)	-1.75 to 0.06	49.50^*b*^	4^*a*^	0.61 (0.68)	-0.73 to 1.98	89.54^*b*^
Creating	Drawing, creating, flower arranging, creating audio/videotape	5	-0.54 (0.56)	-1.64 to 0.54	107.16^*b*^	3^*a*^	1.47 (0.61)	0.25 to 2.67	71.98^*b*^
Thinking	Imagery, goal setting, problem-solving, learning, thought reframing, thought stimulation, learning, gaming, coping	28	-0.11 (0.31)	-0.73 to 0.50	121.10	18	0.47 (0.33)	-0.18 to 1.12	57.39^*b*^
Music	Music listening, singing, playing instruments	14	0.59 (0.39)	-0.16 to 1.37	74.12^*b*^	13	0.89 (0.35)	0.21 to 1.57	49.51^*b*^
Memories	Memories and accomplishments	22	0.14 (0.35)	-0.54 to 0.82	119.98	8	0.07 (0.40)	-0.72 to 0.86	100.75
Senses	Aromatherapy, sensory stimulation through food, smell, sound, and light	13	0.04 (0.36)	-0.66 to 0.73	122.85	9	0.09 (0.38)	-0.66 to 0.85	101.03
Contact	Contact with other residents, therapist, volunteers	45	-0.66 (0.52)	-1.68 to 0.37	75.86^*b*^	27	-0.04 (3.39)	-6.78 to 5.82	101.05
Group-based	Group-based activity, competitive activities	19	-0.21 (0.32)	-0.85 to 0.41	116.25	19	-0.03 (0.32)	-0.65 to 0.59	101.23
Sharing	Sharing memories, thoughts, emotions, and experiences with peers	20	0.13 (0.34)	-0.55 to 0.79	120.54	11	1.07 (0.34)	0.40 to 1.75	-16.05^*b*^
Nature	Activities with animals, greenery, and/or in nature	8	-0.03 (0.63)	-1.26 to 1.22	123.09	7	-0.35 (0.52)	-1.37 to 0.66	90.91^*b*^
Warmth	Giving social support, petting, caretaking	9	-0.22 (0.47)	-1.14 to 0.70	120.52	3^*a*^	-0.07 (0.66)	-1.37 to 1.22	101.00
Positive	Activating, laughing, encouraging, meaningful, hope, acceptance	29	-0.40 (0.31)	-1.02 to 0.22	98.16^*b*^	19	-0.05 (0.33)	-0.71 to 0.61	100.88

All variables are dummy-coded; *N* number of labeled studies per covariate, *ƅ* estimated regression coefficient with negative values indicating better treatment outcomes on severity of DS, *SD* Standard Deviation, *95% CrI* 95% Credible Interval, *DIC* Deviance Information Criterion

^a^Results based on fewer than five included studies per examined covariate might be overfitted and should therefore be taken with caution [64]

^b^Differences in DIC of more than 10 compared to N

## Discussion

### Relative effectiveness of interventions

Compared to CAU, nonpharmacological interventions seemed the most effective in reducing DS, whereas the effects of neurobiological interventions showed almost negligible effects.

Although results of the NMA imply that nonpharmacological interventions should be the preferred approach for reducing DS among NH residents, neurobiological interventions are more common in clinical practice and, in case of major depression, are often advised to be provided alongside nonpharmacological interventions [65]. Compared to CAU, the placebo group was the only intervention type that resulted symptom worsening. This may explain why previous research indicated positive results for neurobiological interventions. Since neurobiological interventions were often compared to placebo interventions, their positive effects found previously might be explained by symptom worsening in the placebo group, rather than by the effectiveness of neurobiological interventions themselves [66].

The beneficial effects of nonpharmacological interventions were similar to those found in previous reviews on managing depression in older adults [67]. With respect to nonpharmacological interventions, most intervention types showed larger effect sizes compared to CAU, suggesting that every action aimed to reduce DS, including being allocated to the waiting list, is better than CAU. This contradicts previously reported findings indicating that the waiting list in psychotherapeutic trials was ‘less effective’ than the CAU group [68]. First possible explanation for this inconsistency with previous research, is that the current research only included participants residing in NHs. It could be that this specific population differs from the general population, for instance due to the type and intensity of care residents usually receive. Second possible explanation for the beneficial effect of the waiting list compared to CAU could be assigned to adherence to assessment procedures in RCT studies among NH residents. This assessment adherence was previously found to reduce DS among participants, even when formal treatment was missing [69]. Therefore, researchers suggested that reduced DS might be explained by informal strategies applied by residents, their relatives, and professional caregivers [19], which could explain the beneficial effect of the waiting list compared to CAU. Third, the beneficial effect of the waiting list compared to CAU may be attributed to the important role of participants’ expectations for change [70]. Being allocated to the waiting list could set up expectations for future treatment and subsequently expectations for improvement of DS in the future.

In conclusion, the aforementioned findings provide further evidence emphasizing the necessity for research to extend beyond investigations employing waiting list, CAU or placebos, prompting a more profound exploration for an enhanced comprehension. For instance, one can compare various disciplines, and explore the contribution of their various components in reducing DS.

### Moderator effects of residents’ factors

The effects of the interventions were moderated by the severity of DS. Results of the analyses showed that studies including participants categorized as having a depression, i.e., residents with more severe DS, reported stronger treatment effects than residents without depression. While the severity of DS moderated treatment effects, there were no changes in the relative effectiveness of intervention types, i.e., the ranking of intervention types. It is not surprising to observe stronger treatment effects in residents with more severe DS. However, the findings regarding the relative effectiveness also suggest that no different interventions are needed with respect to the severity of DS in NH residents. By investigating the moderator effects of components specific to the interventions, we found suggestions that a different strategy in reducing symptoms is indeed necessary, stemming from the multicomponent nature of interventions.

### Moderator effects of components specific to the intervention

Although none of the identified components moderated the effectiveness of interventions, the results of the NMA indicated that some components exhibited larger effect sizes as the severity of DS increases. Results suggested that interventions including health-related components (i.e., daily structure, psychoeducation, food supplements, and healthy food), creativity, contact with others, and positivity (i.e., activating strategies, incorporating hope, encouragement, and focus on happy memories and accomplishments) resulted in stronger effects in residents with more severe DS. These stronger effects suggest that these components might be more important to treat a more severe depression. Interventions including exercise, relaxation, and nature-related components had stronger effects in residents with less severe DS. These stronger effects suggest that these components might be more important for residents with less severe DS, i.e., that these might be important to prevent the onset of depression. Moreover, effect estimators for certain components, such as exercise and health-related components, moved in the opposite direction which may suggest potential symptom worsening in some cases. These results emphasize the necessity to carefully select components in interventions depending on depression severity.

These differences in effects between residents with more severe DS and less severe DS, may be explained by the specific factors or needs of these groups. For instance, major depression in later life was previously found to be more strongly associated with personal vulnerability factors such as cognitive impairment and lack of social support, while minor depression in later life was more strongly associated with adverse life events such as changes in health status [71]. To distract from adverse life events, exercise, relaxation (e.g., breathing exercises), and nature-related components (e.g., contact with flora or fauna) may help to bring one’s attention to the present and may distract from physical and emotional burdens [72, 73]. For personal vulnerability factors in depression, a more persistent and tailored approach providing techniques and skills to cope with these risk factors may be more warranted.

Since tailored interventions consisted mainly of a stepped-care program addressing this issue of carefully selecting interventions depending on depression severity, it is somewhat unexpected that, compared to CAU, tailored interventions were not significant in reducing DS. Most studies did not report on treatment adherence and stakeholder acceptance. Factors such as intrinsic motivation of healthcare professionals [69] may affect treatment pathways within tailored interventions.

Additionally, looking deeper into the individual components may provide more insight. For instance, dividing “social contact with others” into “sharing thoughts, emotions, and memories in a group with peers” and “other activities performed in group,” indicated that the latter might have stronger effects. In addition, contact with therapists, volunteers, and family appears to be more important than contact with peer residents for reducing DS. Moreover, the role of others in activities seem to be more important for residents with more severe DS compared to residents with less severe DS. These findings might be explained by the socioemotional selectivity theory [74], according to which emotional satisfaction in relationships becomes more important for older adults than the size of their network. Since nursing home residents are mostly not fully able to choose fellow residents to interact and share stories with, a lack of emotional satisfaction when performing group activities with peer residents, might have contributed to the insignificant results for reducing DS. In certain group activities, such as sharing personal thoughts and emotions with others, factors such as trust, closeness, and ability to confide in one’s network were previously found to be important protective factors for residents’ mental health [75], and are more likely to be characteristic of individual contact with family and friends. Therefore, the pleasantness and social connectedness experienced during group activities with peer residents that share comparable factors or interests [23, 24] may be protective factors and counterbalance some contradictory findings (i.e., relevance of group activities) of previous research. More research on how to improve emotional satisfaction between residents and better match treatment to the preferences of residents is required.

In conclusion, to tailor treatment depending on depression severity, more insight into the role of the different components in interventions and the facilitating and hindering factors that may contribute to their effectiveness, is needed.

## Strengths and limitations

### Strengths

To the best of our knowledge, this is the first review including all types of depression interventions in NHs that not only considers participant’s level of cognitive impairment but also level of physical dependency, and severity of DS. This resulted in a comprehensive summary of the current evidence and provided greater insight into the importance of accounting for these moderators.

A second strength of this research concerns the integrative method we used to compare the different types of interventions against each other. By comparing both direct and indirect evidence, we were able to contextualize the effectiveness of interventions across the various disciplines, and control for the beneficial effects of interventions in research compared to certain control groups, such as CAU or a placebo control group [76].

A third strength concerns the additional NMRs to weigh heterogeneity within the different types of interventions, suggesting key components in treatments across the various target groups of residents. For example, in both participants with more severe DS and participants with less sever DS, exercise interventions showed significant effects in reducing DS. However, physical exercise in itself had beneficial effects in the group with less severe DS, whereas the opposite effect was found when looking at the group with more severe DS. These contradictions underscore the multicomponent nature of interventions emphasizing the importance of looking beyond the primary intention of interventions.

### Limitations

First, since interventions in studies and eligibility criteria for participants were often limited reported, it was not possible to precisely detect all moderators of interest. Therefore, we were forced to rely on the available data and used aggregated mean scores. This may have led to increased heterogeneity among participants within the different subgroups, and may have affected the results.

Second, the examined moderators were unevenly distributed, and some of the covariates were only represented in a few studies [64]. Therefore, data in this meta-analysis might be overfitted, i.e., false positive results based on too little representative data. Results should be interpreted with caution [77, 78].

Third, although the categorization of interventions was grounded on existing literature, interventions within the various intervention types did differ a lot. For example, green care studies were categorized together based on the definition proposed by Berget et al. [15], and contained both animal-assisted interventions and horticultural therapy (e.g., taking care of a plant)[15]. This variation within intervention types may affect the directness of the meta-analysis [79],suggesting that the evidence of the effectiveness in reducing DS might not be directly linked to the type of intervention [79]. Since adding intervention types may harm the robustness of the network, we countered this diversity of interventions within the intervention types by exploring various components [78]. Results with respect to the ranking of intervention types should, however, be taken with caution.

Finally, we did not control for several other variables such as trial duration, intensity of the interventions, or cultural differences. More research on how this might have contributed to our results is needed.

### Implications for future research

This study elucidates the limitations of the currently available knowledge for depression treatment in NH residents. In future studies, it is needed that the factors of participants are more delineated, and interventions are described in greater detail. Improved descriptions of participants and interventions hold the potential to facilitate research and gain a deeper understanding of the question “what works for whom?”. Moreover, for a more profound exploration, future research should extend beyond the boundaries of one discipline, i.e., beyond investigating specific types of interventions compared solely to CAU or placebos. This can be achieved by working in a more transdisciplinary manner, integrating and systematically investigating insights and components from multiple disciplines to address this complex problem. Finally, other factors such as stakeholders’ acceptance and beliefs are important and may influence the effectiveness of an intervention. These factors are often overlooked and not reported in research, but are very important when it comes to generalizing RCT studies to real world settings [80].

## Conclusions

The continuing and increasing interest in depression interventions in NH residents, specifically for nonpharmacological treatment, was reflected in our results. Using an integrative approach, this meta-analysis emphasizes the significance of not exclusively focusing on the primary intention of interventions but also deliberating and exploring other distinctive factors, including residents’ factors and components specific to the intervention. This study underscores the importance to account for the complexity of interventions, the need for continued transdisciplinary research, and highlights the importance of exploring moderator effects by carefully choosing and constructing interventions adapted to the specific target groups of residents with DS.

### Supplementary Information

Supplementary Material 1.

Supplementary Material 2.

Supplementary Material 3.

Supplementary Material 4.

## Data Availability

The datasets used and/or analysed during the current study available from the corresponding author on reasonable request.

## Abbreviations

NH: Nursing home
DS: Depressive symptoms
RCT: Randomized controlled trials
CAU: Care as usual
NMA: Network meta-analysis
CONSORT: Consolidated standards of reporting trials
EPHPP: Effective public health practice project
GRADE: Grading of recommendations assessment, development and evaluation
MCMC: Markov chain monte carlo
PSRF: Potential scale reduction factor
MD: Mean difference
DIC: Deviance information criterion
PRISMA: Preferred reporting items for systematic reviews and meta-analyses
NMR: Network meta regression

## References

[1] Sivertsen H, Bjørkløf GH, Engedal K, Selbæk G, Helvik AS (2015). A review. Dementia and geriatric cognitive disorders: Depression and Quality of Life in Older Persons.

[2] Yang L, Deng Y-T, Leng Y, Ou Y-N, Li Y-Z, Chen S-D (2023). Depression, depression treatments, and risk of incident dementia: a prospective cohort study of 354,313 participants. Biol Psychiat.

[3] Gilman SE, Sucha E, Kingsbury M, Horton NJ, Murphy JM, Colman I (2017). Depression and mortality in a longitudinal study: 1952–2011. Canadian Medical Association journal = journal de l'Association medicale canadienne (CMAJ).

[4] Sjöberg L, Karlsson B, Atti AR, Skoog I, Fratiglioni L, Wang HX (2017). Prevalence of depression: comparisons of different depression definitions in population-based samples of older adults. J Affect Disord.

[5] Seitz D, Purandare N, Conn D (2010). Prevalence of psychiatric disorders among older adults in long-term care homes: a systematic review. Int Psychogeriatr.

[6] van Zoonen K, Buntrock C, Ebert DD, Smit F, Reynolds CF, Beekman AT, Cuijpers P (2014). Preventing the onset of major depressive disorder: a meta-analytic review of psychological interventions. Int J Epidemiol.

[7] Burley CV, Burns K, Lam BCP, Brodaty H (2022). Nonpharmacological approaches reduce symptoms of depression in dementia: a systematic review and meta-analysis. Ageing Res Rev.

[8] Gramaglia C, Gattoni E, Marangon D, Concina D, Grossini E, Rinaldi C (2021). Non-pharmacological approaches to depressed elderly with no or mild cognitive impairment in long-term care facilities. A systematic review of the literature. Frontiers in public health.

[9] Koch J, Amos JG, Beattie E, Lautenschlager NT, Doyle C, Anstey KJ, Mortby ME (2022). Non-pharmacological interventions for neuropsychiatric symptoms of dementia in residential aged care settings: an umbrella review. Int J Nurs Stud..

[10] Simon GE, Perlis RH (2010). Personalized medicine for depression: can we match patients with treatments. Am J Psychiatry.

[11] Simning A, Simons KV (2017). Treatment of depression in nursing home residents without significant cognitive impairment: a systematic review. Int Psychogeriatr.

[12] Mendes L, Oliveira J, Barbosa F, Castelo-Branco M (2022). A conceptual view of cognitive intervention in older adults with and without cognitive decline—a systemic review. Frontiers in Aging Neuroscience.

[13] Lemmon R, Roseen EJ, Rakel D (2018). Chapter 67 - Chronic Low Back Pain. Integrative Medicine.

[14] Silva R, Abrunheiro S, Cardoso D, Costa P, Couto F, Agrenha C, Apóstolo J (2018). Effectiveness of multisensory stimulation in managing neuropsychiatric symptoms in older adults with major neurocognitive disorder: a systematic review. JBI Database System Rev Implement Rep.

[15] Berget B, Braastad B, Burls A, Elings M, Hadden Y, Haigh R, et al. Green Care: A Conceptual Framework. A report of the Working Group on the Health Benefits of Green Care COST 866, Green care in Agriculture. Loughborough: Loughborough University; 2010 April 2010. Report No.: Loughborough University.

[16] NICE guideline (2022). Depression in adults: treatment and management. British National Formulary.

[17] Jongenelis K, Pot AM, Eisses AM, Beekman AT, Kluiter H, Ribbe MW (2004). Prevalence and risk indicators of depression in elderly nursing home patients: the AGED study. J Affect Disord.

[18] Gaugler JE, Duval S, Anderson KA, Kane RL (2007). Predicting nursing home admission in the U.S: a meta-analysis. BMC Geriatr..

[19] Knippenberg IAH, Leontjevas R, Stoyanov S, Persoon A, Verboon P, Vermeulen H (2022). Informal antidepressant strategies for nursing home residents: two group concept mapping studies. Aging Ment Health.

[20] McLaughlin JA, Jordan GB (2015). Using Logic Models. Handbook of Practical Program Evaluation.

[21] Pinquart M (1998). Wirkungen psychosozialer und psychotherapeutischer Interventionen auf das Befinden und das Selbstkonzept im höheren Erwachsenenalter-Ergebnisse von Metaanalysen. Z Gerontol Geriatr.

[22] Cuijpers P, van Straten A, Warmerdam L (2008). Are individual and group treatments equally effective in the treatment of depression in adults? A meta-analysis. The. European J Psychiatr.

[23] Diegelmann M, Jansen CP, Wahl HW, Schilling OK, Schnabel EL, Hauer K (2018). Does a physical activity program in the nursing home impact on depressive symptoms? A generalized linear mixed-model approach. Aging Ment Health.

[24] Knippenberg IAH, Reijnders JSAM, Gerritsen DL, Leontjevas R (2019). The association between specific activity components and depression in nursing home residents: the importance of the social component. Aging Ment Health.

[25] Terry PC, Karageorghis CI, Curran ML, Martin OV, Parsons-Smith RL (2020). Effects of music in exercise and sport: a meta-analytic review. Psychol Bull.

[26] Gómez-Gallego M, Gómez-Gallego JC, Gallego-Mellado M, García-García J (2021). Comparative efficacy of active group music intervention versus group music listening in Alzheimer's Disease. Int J Environ Res Pub Health..

[27] Pereira APS, Marinho V, Gupta D, Magalhães F, Ayres C, Teixeira S (2019). Music therapy and dance as gait rehabilitation in patients with Parkinson disease: a review of evidence. J Geriatr Psychiatry Neurol.

[28] Tsoi KKF, Chan JYC, Ng Y, Lee MMY, Kwok TCY, Wong SYS (2018). Receptive music therapy is more effective than interactive music therapy to relieve behavioral and psychological symptoms of dementia: a systeatice review and meta-analysis. J American Med Direct Assoc.

[29] Janata P, Tomic ST, Rakowski SK. Characterization of music-evoked autobiographical memories. *Memory*. 2007;15(8):845–60. 10.1080/09658210701734593 17965981

[30] Williams SE, Ford JH, Kensinger EA (2022). The power of negative and positive episodic memories. Cogn Affect Behav Neurosci.

[31] Westerhof GJ, Korte J, Eshuis S, Bohlmeijer ET (2018). Precious memories: a randomized controlled trial on the effects of an autobiographical memory intervention delivered by trained volunteers in residential care homes. Aging Ment Health.

[32] McKenzie JE, Brennan SE, Ryan RE, Thomson HJ, Johnston RV, Thomas J. Chapter 3: Defining the criteria for including studies and how they will be grouped for the synthesis. In: Higgins JPT TJ, Chandler J, Cumpston M, Li T, Page MJ, Welch VA (editors), editor. Cochrane Handbook for Systematic Reviews of Interventions version 63 February 2022 ed: Cochrane; 2022. www.training.cochrane.org/handbook.

[33] Hariton E, Locascio JJ (2018). Randomised controlled trials - the gold standard for effectiveness research: study design: randomised controlled trials. BJOG.

[34] Lefebvre C, Glanville J, Briscoe S, Featherstone R, Littlewood A, Marshall C, et al. Chapter 4: Searching for and selecting studies. In: Higings JPT, Thomas J, Chandler J, Cumpston M, Li T, Page MJ, et al., editors. Cochrane Handbook for Systematic Reviews of Interventions version 63 February 2022 ed: Cochrane; 2022. www.training.cochrane.org/handbook.

[35] Chen SC, Jones C, Moyle W (2018). Social robots for depression in older adults: a systematic review. J Nurs Scholarsh.

[36] Folkerts A, Roheger M, Franklin J, Middelstädt J, Kalbe E (2017). Cognitive interventions in patients with dementia living in long-term care facilities: systematic review and meta-analysis. Arch Gerontol Geriatr.

[37] Jain B, Syed S, Hafford-Lechfield T, O'Farell-Pearce S (2020). Dog-assisted interventions and outcomes for older adults in residential long-term care facilities: a systematic review and meta-analysis. Int J Older People Nurs.

[38] Dennet L. Randomized Controlled Trials/Controlled Clinical Trials: A cut and paste Search Strategy adapted from CADTH for Ovid PsycINFO2020. Available from: https://docs.google.com/document/d/1g7vXZz2CAAqZpbHoOFxxiCVDdCW6bRsDuvqYZDiMH5s/edit.

[39] Glanville J, Dooley G, Wisniewski S, Foxlee R, Noel-Storr A (2019). Development of a search filter to identify reports of controlled clinical trials within CINAHL plus. Health Info Libr J.

[40] Ouzzani M, Hammady H, Fedorowicz Z, Elmagarmid A (2016). Rayyan - a web and mobile app for systematic reviews. Syst Rev.

[41] Schulz KF, Altman DG, Moher D, CONSORT Group (2010). CONSORT 2010 Statement: updated guidelines for reporting parallel group randomised trials. Ann Intern Med.

[42] Thomas BH, Ciliska D, Dobbins M, Micucci SA. A process for systematically reviewing the literature: Providing the research evidence for public health nursing interventions. *Worldviews on evidence-based nursing*. 2004;1(3):176-84. 10.1111/j.1524-475X.2004.04006.x 17163895

[43] Egger M, Smith GD, Schneider M, Minder C (1997). Bias in meta-analysis detected by a simple. Graphical test. BMJ.

[44] Meader N, King K, Llewellyn A, Norman G, Brown J, Rodgers M (2014). A checklist designed to aid consistency and reproducibility of GRADE asessments: development and pilot validation. Syst Rev..

[45] Puhan MA, Schünemann H, Murad MH, Li T, Brignadello-Peterson R, Singh JA (2014). A GRADE working group approach for rating the quality of treatment effect estimates from network meta-analysis. BMJ..

[46] Salanti G, Del Giovane C, Chaimani A, Caldwell DM, Higgins JP (2014). Evaluating the quality of evidence from a network meta-analysis. PLoS ONE.

[47] RStudio Team (2022). RStudio: Integrated Development Environment for R. 2022.2.3.492 ed.

[48] Harrer M, Cuijpers P, Furukawa TA, Ebert DD (2021). Doing Meta-Analysis with R: A hands-on Guide.

[49] Higgings JPT, Li T, Deeks JJ. Chapter 6: Choosing effect measures and computing estimates of effect. In: Higgins JPT, Thomas J, Chandler J, Cumpston M, Li T, Page MJ, Welch VA, editors. Cochrane Handbook for Systematic Reviews of Interventions version 63 February 2022 ed. https://www.training.cochrane.org/handbook: Cochrane; 2021.

[50] Franchini AJ, Dias S, Ades AE, Jansen JP, Welton NJ (2012). Accounting for correlation in network meta-analysis with multi-arm trials. Res Synth Methods.

[51] Brooks SP, Gelman A (1998). General methods for monitoring convergence of iterative simulations. J Comput Graph Stat.

[52] Tonin FS, Rotta I, Mendes AM, Pontarolo R (2017). Network meta-analysis: a technique to gather evidence from direct and indirect comparisons. Pharmacy pract..

[53] Dias S, Welton NJ, Caldwell DM, Ades AE (2010). Checking consistency in mixed treatment comparison meta-analysis. Stat Med.

[54] Gelman A, Carlin JB, Stern HS, Dunson DB, Vehtari A, Rubin DB. Bayesian data analysis. New York: Chapman and Hall/CRC; 2013.

[55] Lunn D, Jackson C, Best N, Thomas A, Spiegelhalter D. The BUGS Book – A Practical Introduction to BayesianAnalysis. New York: Chapman and Hall/CRC; 2012.

[56] Page MJ, McKenzie JE, Bossuyt PM, Boutron I, Hoffmann TC, Mulrow CD, et al. The PRISMA 2020 statement: an updated guideline for reporting systematic reviews. Bmj. 2021;372:n71. 10.1136/bmj.n71 PMC8005924 33782057

[57] Kim H-S, Kang J-S (2021). Effect of a group music intervention on cognitive function and mental health outcomes among nursing home residents: A randomized controlled pilot study. Geriatr Nurs.

[58] Chu H-Y, Chen M-F, Tsai C-C, Chan H-S, Wu T-L (2019). Efficacy of a horticultural activity program for reducing depression and loneliness in older residents of nursing homes in Taiwan. Geriatr Nurs.

[59] Erdal A, Flo E, Aarsland D, Ballard C, Slettebo DD, Husebo BS (2018). Efficacy and safety of analgesic treatment for Depression in People with Advanced Dementia: Randomised, Multicentre, Double-Blind, Placebo-Controlled Trial (DEP.PAIN.DEM). Drugs Aging.

[60] Oslin DW, Ten Have TR, Streim JE, Datto CJ, Weintraub D, DiFilippo S, Katz IR (2003). Probing the safety of medications in the frail elderly: Evidence from a randomized clinical trial of sertraline and venlafaxine in depressed nursing home residents. J Clin Psychiatry.

[61] Sánchez A, Maseda A, Marante-Moar MP, de Labra C, Lorenzo-López L, Millán-Calenti JC (2016). Comparing the effects of multisensory stimulation and individualized music sessions on elderly people with severe dementia: a randomized controlled trial. J Alzheimers Dis.

[62] Veleva BI, Caljouw MAA, van der Steen JT, Mertens BJA, Chel VGM, Numans ME (2020). The effect of ultraviolet B irradiation compared with oral vitamin D supplementation on the well-being of nursing home residents with dementia: a randomized controlled trial. Int J Environ Res Public Health..

[63] Werner J, Wosch T, Gold C (2016). Effectiveness of group music therapy versus recreational group singing for depressive symptoms of elderly nursing home residents. Aging Ment Health.

[64] Geissbühler M, Hincapié CA, Aghlmandi S, Zwahlen M, Jüni P, da Costa BR (2021). Most published meta-regression analyses based on aggregate data suffer from methodological pitfalls: a meta-epidemiological study. BMC Med Res Methodol.

[65] Gartlehner G, Wagner G, Matyas N, Titscher V, Greimel J, Lux L (2017). Pharmacological and non-pharmacological treatments for major depressive disorder: review of systematic reviews. BMJ Open.

[66] Kirsch I, Deacon BJ, Huedo-Medina TB, Scoboria A, Moore TJ, Johnson BT. Initial severity and antidepressant benefits: a meta-analysis of data submitted to the Food and Drug Administration PLoS medicine. 2008;5(2):e45. 10.1371/journal.pmed.0050045 PMC2253608 18303940

[67] Farah WH, Alsawas M, Mainou M, Alahdab F, Farah MH, Ahmed AT, et al. Non-pharmacological treatment of depression: a systematic review and evidence map Evidence-based medicine. 2016;21(6):214-21. 10.1136/ebmed-2016-110522 27836921

[68] Furukawa TA, Noma H, Caldwell DM, Honyashiki M, Shinohara K, Imai H (2014). Waiting list may be a nocebo condition in psychotherapy trials: a contribution from network meta-analysis. Acta Psychiatr Scand.

[69] Leontjevas R, Gerritsen DL, Smalbrugge M, Teerenstra S, Vernooij-Dassen MJ, Koopmans RT (2013). A structural multidisciplinary approach to depression management in nursing-home residents: a multicentre, stepped-wedge cluster-randomised trial. Lancet.

[70] Kazdin AE (2015). Treatment as usual and routine care in research and clinical practice. Clin Psychol Rev.

[71] Wu CS, Yu SH, Lee CY, Tseng HY, Chiu YF, Hsiung CA (2017). Prevalence of and risk factors for minor and major depression among community-dwelling older adults in Taiwan. Int Psychogeriatr.

[72] Levit-Binnun N, Arbel K, Dorjee D (2021). The mindfulness map: a practical classification framework of mindfulness practices, associated intentions, and experiential understandings. Front Psychol.

[73] Reniers PWA, Declercq IJN, Hediger K, Enders-Slegers MJ, Gerritsen DL, Leontjevas R (2022). The role of pets in the support systems of community-dwelling older adults: a qualitative systematic review. Aging Ment Health.

[74] Carstensen LL (1992). Social and emotional patterns in adulthood: support for socioemotional selectivity theory. Psychol Aging.

[75] Chipps J, Jarvis MA (2016). Social capital and mental well-being of older people residing in a residential care facility in Durban, South Africa. Aging Ment Health.

[76] Mohr DC, Ho J, Hart TL, Baron KG, Berendsen M, Beckner V (2014). Control condition design and implementation features in controlled trials: a meta-analysis of trials evaluating psychotherapy for depression. Transl Behav Med.

[77] Ying X (2019). An overview of overfitting and its solutions. J Phys: Conf Ser.

[78] Deeks JJ, Higgins JPT, Altman DG, Higgins JPT, Thomas J, Chandler J, Cumpston M, Li T, Page MJ, Welch VA (2022). Chapter 10: Analysing data and undertaking meta-analyses. Cochrane Handbook for Systematic Reviews of Interventions version 63 version 6.3. February 2022 ed2022.

[79] Guyatt GH, Oxman AD, Kunz R, Woodcock J, Brozek J, Helfand M (2011). GRADE guidelines: 7. Rating the quality of evidence–inconsistency. J Clin Epidemiol.

[80] Ormel J, Hollon SD, Kessler RC, Cuijpers P, Monroe SM (2022). More treatment but no less depression: the treatment-prevalence paradox. Clin Psychol Rev.

