# Supplementary material for: A bayesian network meta-analysis to explore modifying factors in randomized controlled trials: what works for whom to reduce depression in nursing home residents?
Source: BMC Geriatr. 2024 Jun 14;24:518. doi: 10.1186/s12877-024-05117-8 (PMC11177425; doi:10.1186/s12877-024-05117-8)
Supplement: Supplementary file 1 — Supplementary Material 1. [file 12877_2024_5117_MOESM1_ESM.docx]

**Additional File 1 - Current state of the literature**

A wide range of interventions can be applied to reduce depressive symptoms (DS) in nursing home (NH) residents. This appendix gives a brief overview of the current state of the literature. In accordance with Koch and colleagues’ umbrella review (2022), the following intervention types could be distinguished in the literature.

# Cognitive Interventions

Cognitive interventions include “the variety of therapeutic approaches designed to address psychological problems at the cognitive (conscious mind) level” (1). Mendes and colleagues’ review (2022) distinguishes eight types of cognitive interventions, namely, compensatory cognitive training, cognitive remediation, enrichment, cognitive activation, brain training, cognitive stimulation, cognitive training, and cognitive rehabilitation. A few well-known examples used within a psychotherapeutic context to reduce DS in NH residents include cognitive behavioral therapy and pleasant activity scheduling. Both interventions are aimed to change thinking and behavioral patterns, which may result in positive effects on mental health and DS. Although the results of the effectiveness of cognitive interventions on DS are promising, results are difficult to generalize due to the limited currently-available evidence, and heterogeneity between studies and included participants, for instance people with and without cognitive impairment (2, 3).

# Exercise Interventions

Lemmon & Roseen (2018) describe exercise interventions as the “broad range of techniques that are used to improve strength, coordination, flexibility, range of motion, endurance and aerobic capacity”(4). A few examples used within the context to reduce DS in NH residents include band resistance exercise and aerobics. Although exercise interventions are mostly functional and aimed to improve activities of daily living (5), researchers found a positive effect of exercise on reducing DS (6). However, it remains unclear whether these effects are physical, neurobiological, or, rather, psychosocial in nature. For example, some researchers hypothesized that exercise produces certain biological effects and, consequently, a stress-reducing and mood-enhancing effect (7), whereas other researchers highlighted important psychosocial mechanisms in physical activities such as social interaction, improved physical self-perception, and transferable skills to cope with challenges (8).

# Sensory Stimulation

Sensory stimulation was previously defined as interventions "aimed to stimulate the primary senses to achieve a balance between sensory-stimulating and sensory-calming activities” which may result in an “atmosphere of trust and relaxation” (9). Music therapy, aromatherapy, multisensory therapy, and massage are some examples of these interventions. Results of the effectiveness of these interventions on DS are rather mixed due to inconsistent approaches in interventions (10).

# Green Care

Based on Berget and colleagues’ (2010) definition, green care interventions include a “diverse set of interventions using nature and natural environment as a framework in which to create these approaches” (11). A few common examples of green care interventions are care farming, animal-assisted therapy, and horticulture therapy. Reviewers suggested that behavioral activation, social support, and improved self-efficacy within green care interventions could be the potential key elements of the positive effects of green care interventions in reducing DS (12). However, more research on underlying mechanisms is needed, especially with respect to this specific group of NH residents.

# Neurobiological Interventions

In addition to Koch et al. and colleagues’ review (2022), the present review also includes neurobiological interventions. Although there may be some overlap, these interventions could be distinguished from others based on their primary goal, which is the direct targeting of neurobiological mechanisms. Neurobiological interventions include psychotropic drugs (e.g., Selective Serotonin Reuptake Inhibitors [SSRIs]), light therapy, nutritional additives, and electroconvulsive therapy. The currently-available evidence of these neurobiological interventions in reducing depressive symptoms is rather limited and inconclusive (13).

# Tailored Interventions

Tailored interventions are adapted to the specific needs of individual residents using the “least restrictive and least costly intervention that will be effective for a person’s presenting problems” (14). The following steps could be distinguished: 1) depression screening and monitoring, encouraging activities that promote health (e.g., pleasant and meaningful activities), and psychoeducation, 2) high- or low-intensity psychotherapeutic and psychosocial approaches (whether or not combined with medication), 3) medication combined with psychotherapeutic (14) approaches, 4) multi-professional combined treatment. The effectiveness of this stepped-care approach is supported by research (15) and encouraged in different guidelines for depression treatment (14, 16, 17). However, tailoring treatment is a time-consuming process, which could be challenging in daily practice.

# References

1. Mendes L, Oliveira J, Barbosa F, Castelo-Branco M. A Conceptual View of Cognitive Intervention in Older Adults With and Without Cognitive Decline—A Systemic Review. Frontiers in Aging Neuroscience. 2022;3:844725.

2. Burley CV, Burns K, Lam BCP, Brodaty H. Nonpharmacological approaches reduce symptoms of depression in dementia: A systematic review and meta-analysis. Ageing research reviews. 2022;79:101669.

3. Gramaglia C, Gattoni E, Marangon D, Concina D, Grossini E, Rinaldi C, et al. Non-pharmacological Approaches to Depressed Elderly With No or Mild Cognitive Impairment in Long-Term Care Facilities. A Systematic Review of the Literature. Frontiers in public health. 2021;9:685860.

4. Lemmon R, Roseen EJ. Chapter 67 - Chronic Low Back Pain. In: Rakel D, editor. Integrative Medicine (Fourth Edition): Elsevier; 2018. p. 662-75.e3.

5. Liu C-j, Shiroy D, M. , Jones L, Y. , Clark D, O. . Systematic review of functional training on muscle strength, physical functioning, and activities of daily living in older adults. European Review of Aging and Physical Activity (EURAPA). 2014;11:95-106.

6. Cooney GM, Dwan K, Greig CA, Lawlor DA, Rimer J, Waugh FR, et al. Exercise for depression. The Cochrane database of systematic reviews. 2013;9:CD004366.

7. Stammes R, Spijker J. Fysieke training bij depressie; een overzicht. Tijdschrift voor psychiatrie 51. 2009;11:821-30.

8. Kandola A, Ashdown-Franks G, Hendrikse J, Sabiston CM, Stubbs B. Physical activity and depression: Towards understanding the antidepressant mechanisms of physical activity. Neuroscience and biobehavioral reviews. 2019;107:525-39.

9. Silva R, Abrunheiro S, Cardoso D, Costa P, Couto F, Agrenha C, et al. Effectiveness of multisensory stimulation in managing neuropsychiatric symptoms in older adults with major neurocognitive disorder: a systematic review. JBI database of systematic reviews and implementation reports. 2018;16(8):1663-708.

10. Koch J, Amos JG, Beattie E, Lautenschlager NT, Doyle C, Anstey KJ, et al. Non-pharmacological interventions for neuropsychiatric symptoms of dementia in residential aged care settings: An umbrella review. International Journal of Nursing Studies. 2022:104187.

11. Berget B, Braastad B, Burls A, Elings M, Hadden Y, Haigh R, et al. Green Care: A Conceptual Framework. A report of the Working Group on the Health Benefits of Green Care COST 866, Green care in Agriculture. Loughborough: Loughborough University; 2010 April 2010. Report No.: Loughborough University.

12. Salomon RE, Salomon AD, Beeber LS. Green Care as Psychosocial Intervention for Depressive Symptoms: What Might Be the Key Ingredients. Journal of the American Psychiatric Nurses Association. 2018;24(3):199-208.

13. Simning A, Simons KV. Treatment of depression in nursing home residents without significant cognitive impairment: a systematic review. International psychogeriatrics. 2017:209-26.

14. NICE guideline. Depression in adults: treatment and management. British National Formulary; 2022 29 June 2022.

15. Leontjevas R, Gerritsen DL, Smalbrugge M, Teerenstra S, Vernooij-Dassen MJ, Koopmans RT. A structural multidisciplinary approach to depression management in nursing-home residents: a multicentre, stepped-wedge cluster-randomised trial. Lancet. 2013;381(9885):2255-64.

16. Declercq T, Habraken H, van den Ameele H, Callens J, De Lepeleire J, Cloetens H. Richtlijn voor goede medische praktijkvoering: Depressie bij volwassenen. Antwerpen: Domus Medica vzw; 2017.

17. Gerritsen D, Leontjevas R, Ketelaar N, Derksen E, Koopmans R, Smalbrugge M. Databank interventies langdurende zorg: beschrijving “Doen bij Depressie”. Utrecht: Vilans; 2014.
