# Supplementary material for: A bayesian network meta-analysis to explore modifying factors in randomized controlled trials: what works for whom to reduce depression in nursing home residents?
Source: BMC Geriatr. 2024 Jun 14;24:518. doi: 10.1186/s12877-024-05117-8 (PMC11177425; doi:10.1186/s12877-024-05117-8)

# Additional File 2 – Study selection and data extraction

This appendix contains information with respect to the selection of studies and the collection of data of interest, i.e., selection criteria, search strategy, protocol for data-extraction, and decision tree.

## Study Selection

### Table 2.1. Inclusion- and exclusion criteria per PICO element

|  | Included Studies | Excluded Studies |
| --- | --- | --- |
| PARTICIPANTS | - Nursing Home Residents - Residential Care Facilities - Assisted Living - Long Term Care Residents | - other setting: outpatients, community -dwelling, noninstitutionalized, hospitalized, general practice, newly admitted - Based on age: youth, adolescents, children - Bipolar/schizophrenic/ treatment resistant |
| INTERVENTION | - Primary aimed to reduce depressive symptoms | - No exclusion criteria |
| CONTROL GROUP | - CAU - Waiting List - Placebo - Other Intervention | - No exclusion criteria |
| OUTCOME | - Extent of DS= primary outcome - Depression on symptom level, measured with a standardized measurement tool - Proxy-rated - Self-rated | - Depression was not the primary outcome (e.g., pain, physical health, cognitive functioning) - Depression outcome was not measured on symptom level (e.g., incidence rate based on DSM-V, reduction in antidepressant drug use) - Hedges’ *g* could not be calculated |
| STUDY DESIGN | - Randomized Controlled Trial - Clustered RCT (on nursing home, ward, floor) - Purposive sampling/Quasi experimental (only if this technique was used to select participants from one of the intended subgroups) | - Review, meta-analysis - Poster, abstracts, books, dissertations - Retrospective, secondary analysis, extension studies, follow-up studies - Observational studies, cross-sectional analysis, cohort studies, one-armed trials - Quasi-experimental - Convenience/purposive sampling (other than intended subgroups) - Nonrandomized |

### Table 2.2. Search Strategy

*Pubmed*

|  | Search string |
| --- | --- |
| 1: Nursing home | “nursing homes”[MeSH Terms] OR "homes for the aged"[MeSH Terms] OR "long-term care"[MeSH Terms] OR "nursing home*"[Title/Abstract] OR "home for the aged"[Title/Abstract] OR “Homes for the aged"[Title/Abstract] OR "long term care*"[Title/Abstract] |
| 2: Depression | "depression"[MeSH Terms] OR "depressive disorder, major"[MeSH Terms] OR "depressive disorder"[MeSH Terms] OR “depress*"[Title/Abstract] |
| 3: Study Design (Lefebvre, 2022) | ("randomized controlled trial"[Publication Type] OR "drug therapy"[MeSH Subheading] OR "randomized"[Title/Abstract] OR "randomised"[Title/Abstract] OR "placebo"[Title/Abstract] OR "randomly"[Title/Abstract] OR "trial"[Title/Abstract] OR "groups"[Title/Abstract]) NOT ("animals"[MeSH Terms] NOT "humans"[MeSH Terms]) |
| 1 AND 2 AND 3 AND 4 | #1 AND #2 AND #3 |

*Embase*

|  | Search string |
| --- | --- |
| 1: Nursing home | Nursing home/ OR long-term care/ OR home for the aged/ OR nursing home patient/  OR (Nursing home* OR Home? For the aged OR Long term care*).ti,ab,kw |
| 2: Depression | Exp depression / OR (depress*).ab,ti,kw |
| 3: Study Design (Lefebvre, 2022) | (exp Randomized controlled trial/ OR random$.ti,ab. OR randomization/ OR intermethod comparison/ OR placebo.ti,ab. OR (compare or compared or comparison).ti. OR ((evaluated or evaluate or evaluating or assessed or assess) and (compare or compared or comparing or comparison)).ab. OR ((double or single or doubly or singly) adj (blind or blinded or blindly)).ti,ab. OR double blind procedure/ OR parallel group$1.ti,ab. OR ((assign$ or allocation) adj5 (alternate or group$1 or intervention$1 or patient$1 or subject$1 or participant$1)).ti,ab. OR (assigned or allocated).ti,ab. OR (volunteer or volunteers).ti,ab. OR human experiment/ OR trial.ti.) NOT (((random$ adj sampl$ adj7 ("cross section$" or questionnaire$1 or survey$ or database$1)).ti,ab. Not (comparative study/ or randomi?ed controlled.ti,ab. Or randomly assigned.ti,ab.)) OR (Cross-sectional study/ not (randomized controlled trial/ or randomi?ed controlled.ti,ab. Or control group$1.ti,ab.)) OR (((case adj control$) and random$) not randomi?ed controlled).ti,ab. OR (Systematic review not (trial or study)).ti. OR (nonrandom$ not random$).ti,ab. OR "Random field$".ti,ab. OR (random cluster adj3 sampl$).ti,ab. OR ((review.ab. and review.pt.) not trial.ti.) OR ("we searched".ab. and (review.ti. or review.pt.)) OR "update review".ab. OR (databases adj4 searched).ab. OR ((rat or rats or mouse or mice or swine or porcine or murine or sheep or lambs or pigs or piglets or rabbit or rabbits or cat or cats or dog or dogs or cattle or bovine or monkey or monkeys or trout or marmoset$1).ti. and animal experiment/) OR (Animal experiment/ not (human experiment/ or human/))) |
| 4 Limits | limit 3 to conference abstract status |
| 5 Combined search | 1 AND 2 AND 3 |
| 6 Limited search | 5 NOT 4 |

*PsycINFO*

|  | Search string |
| --- | --- |
| 1: Nursing home | Exp Nursing homes/ OR long-term care/  OR (Nursing home* OR Home? For the aged OR Long term care*).ti,ab,id |
| 3: Depression | exp "Depression (Emotion)"/ or exp Major Depression/ or depress*.ab,ti,id |
| 4: Study Design (Health, 2020) | treatment effectiveness evaluation/ or exp randomized controlled trials/ or placebo/ or followup studies/ or (placebo* or random* or comparative stud* or Allocated* or trial* or (research adj4 design) or (evaluat* adj4 stud*) or (phase adj3 (III or "3") adj3 (study or studies or trial*)) or (prospectiv* adj4 stud*) or ((singl* or doubl* or trebl* or tripl*) adj4 (blind* or mask*))).ti,ab,id |
| 1 AND 2 AND 3 AND 4 | 1 AND 2 AND 3 |

*Cinahl*

|  | Search string |
| --- | --- |
| 1: Nursing home | (MH "Nursing Homes+") OR (MH "Nursing Home Patients")  OR (MH "Long Term Care") *OR (TI “Nursing home*” OR “Home# for the aged” OR “Long term care*”) OR (AB “Nursing home*” OR “Home# for the aged” OR “Long term care*”)* |
| 2: Depression | (MH "Depression+") OR *(TI depress*) OR (AB depress*)* |
| 3: Study Design (Glanville J, 2019b) | ( (MH “randomized controlled trials”) OR (MH “double-blind studies”) OR (MH “single-blind studies”) OR (MH “random assignment”) OR (MH “pretest-posttest design”) OR (MH “cluster sample”) OR (TI randomised OR randomized) OR (AB random*) OR (TI trial) OR ((MH “sample size”) AND (AB (assigned OR allocated OR control))) OR (MH “placebos”) OR (PT randomized controlled trial) OR (AB control W5 group) OR (MH “crossover design”) OR (MH “comparative studies”) OR (AB cluster W3 RCT) ) NOT (((MH “animals+”) OR (MH “animal studies”) OR (TI animal model*)) NOT (MH "human"))) |
| 1 AND 2 AND 3 | S1 AND S2 AND S3 |

*Cochrane*

|  | Search string |
| --- | --- |
| 1: Nursing home | [mh "nursing homes"] OR [mh "homes for the aged"] OR [mh "long-term care"] OR ("Nursing home" OR “Nursing homes" OR "Home for the aged" OR "Homes for the aged" OR "Long term care"):ti,ab,kw |
| 2: Depression | [mh “depression”] OR [mh "depressive disorder, major"] OR [mh "depressive disorder"] OR (depress*):ti,ab,kw |
|  | / |
| 1 AND 2 AND 3 | #1 AND #2 |

*Web Of Science*

|  | Search string |
| --- | --- |
| 1: Nursing home | TS = ("Nursing home*" OR "Home$ for the aged" OR "Long term care*") |
| 2: Depression | TS = depress* |
| 3: Study Design (Glanville J, 2019b) | TS = (randomised OR randomized OR randomisation OR randomization OR placebo* OR trial* OR (random* AND (allocat* OR assign*)) OR (blind* AND (single OR double OR treble OR triple))) |
| 1 AND 2 AND 3 | #1 AND #2 AND #3 |

## Protocol for data extraction

*Note: questions indicated with “*” were derived from the EPHPP tool (2009)*

1. **DECISION**

Decision? Include/Exclude/Maybe – Reason Exclusion

*=> If excluded: give reason: i.e., Study Design / Participants /Outcome/ Analysis*

1. **STUDY DESIGN**

**Design**

Trial Design? Yes/No

*Design Description?

1 Randomized controlled trial

2 Stratified Randomized Controlled Trial

3 Clustered Randomized Controlled Trial

4 Purposive/convenience sampling based on residents characteristic (dependency/dementia) or components specific to the intervention (e.g., being able to communicate if participants took part of a discussion group; not allergic to animals in AAI)

~~5 Case-control~~ => exclusion

~~6 Cohort (one group pre + post (before and after))~~ => exclusion

~~7 Interrupted time series~~ => exclusion

~~8 Can’t tell~~ => exclusion

*=> In case of 5 to 8: exclude article (reason exclusion = Study Design: …)*

Measurement Moments? Pre/post/follow-up? *=> results included in the meta-analysis*

Trial Duration? Number of weeks *=> duration of the treatment, not follow-up period, in weeks*

**Randomisation**

Description Randomisation? Describe process of randomization

*Was the study described as randomized controlled trial? Yes/No

*Was the method of randomization described? Yes/No

*=> yes if authors describe any method used to generate a random allocation sequence*

*If yes, was the method appropriate? Yes/No/Can’t tell

*=> randomization sequence allowed each study participant to have the same chance of receiving each intervention and the investigators could not predict which intervention was next. Examples of appropriate approaches include assignment of subjects by a central office unaware of subject characteristics, or sequentially numbered, sealed, opaque envelopes.*

*Use only “Can’t tell” when nothing is mentioned*

1. **METHOD**

**Intervention Description**

Nursing Home Residents?

1. Nursing Home Residents
2. Residential Care Facilities
3. Assisted Living Facilities
4. Long Term Care Residents

*=> If none is applicable (e.g., Hospitalization, community dwelling,…)*

*=> Exclusion: Participants:…*

How Many Nursing Homes? Number of nursing homes included in the study

Continent? Africa/America/Asia/Australia/Europe

Location? Country

Type Intervention? Basic/Psychotherapeutic/ Neurobiological

Kind Intervention?

Cognitive Interventions/ Exercise/Green Care /Psychosocial interventions/ Sensory Stimulation/Tailored Interventions/Pet-robots/Reminiscence

Intervention Description

Describe the intervention (e.g. Reminiscence: ….)

Control Group:

Cognitive Interventions/ Exercise/Green Care /Psychosocial interventions/ Sensory Stimulation/Tailored Interventions/Pet-robots/Reminiscence

Description Control:

Describe the control group (e.g. Reminiscence: ….)

**Outcomes**

Was depression Primary Outcome? Yes/No

*=> If “No” => Exclusion: Outcome:…*

Instrument used for Depression Used instrument for depression

Validated? Yes/No

*=> If “No” => Exclusion: Outcome:…*

Other Outcomes? Secondary Outcomes

*Were data collection tools shown to be valid? Yes/No/Can’t tell

*Were data collection tools shown to be reliable? Yes/No/Can’t tell

*=> Only “Yes” when Cronbach’s Alpha is mentioned and is >.69; If not mentioned: “Can’t tell”*

**Blinding**

*Was (were) the outcome assessor(s) aware of the intervention or exposure status of participants?

Yes/No/Can’t tell

*(Q1) Assessors should be described as blinded to which participants were in the control and intervention groups. The purpose of blinding the outcome assessors (who might also be the care providers) is to protect against detection bias.*

*Were the study participants aware of the research question?

Yes/No/Can’t tell

*(Q2) Study participants should not be aware of (i.e. blinded to) the research question. The purpose of blinding the participants is to protect against reporting bias.*

*Indicate “yes” if words like informed, detailed explanation of the study… were used.*

1. **INTERVENTION CHARACTERISCTICS**

*Components specific to the intervention (adapted from Knippenberg et al., 2022)*

**Body**

- Exercise: i.e., small motor skills, coordination, ROM (flexibility, stretching, postural re-education), strength (resistance), balance, aerobic (walking), dance, ADL-training, yoga, tai chi
- Touch: i.e., massage, touching animals
- Relaxation: i.e., mindfulness, breathing exercises, yoga, tai chi, massage
- Health: i.e., psychoeducation, structure, nutrition

**Mind**

- Creativity: i.e., drawing, creating, flower arranging, creating audio/videotape
- Thinking: i.e., imagery, goal setting, problem-solving, learning, thought reframing, thought stimulation, learning, gaming, coping
- Music: i.e., music listening, singing, playing instruments
- Memories: i.e., memories and accomplishments
- Sensory (visual, auditory, tactile, and olfactory): i.e., aromatherapy, sensory stimulation through food, smell, sound, and light

**Bonding**

- Group: i.e., group activity, competitive activities, individual contact
- Sharing: i.e., sharing thoughts, emotions, and experiences with peers, sharing memories
- Society: i.e., going out on a trip, contact with society
- Nature : i.e., animal, nature

**Other/positivity**

- Warmth: i.e., giving social support, petting, caretaking
- Positivity: i.e., activating, laughing, encouraging, meaningful, hope, acceptance

1. **STATISTICAL METHOD**

**Statistical Method**

*Is the analysis performed by intervention allocation status (i.e. intention to treat) rather than the

actual intervention received? Yes/No/Can’t tell

*=>An intention-to-treat analysis is one in which all the participants in a trial are analyzed according to the intervention to which they were allocated, whether they received it or not. Intention-to-treat analyses are favoured in assessments of effectiveness as they mirror the noncompliance and treatment changes that are likely to occur when the intervention is used in practice, and because of the risk of attrition bias when participants are excluded from the analysis.*

**Reach**

Sample Pool description? Yes/No

*=> Description of population from which sample was drawn*

Sample Description? Yes/No *(only yes if more then 3 variables were described)*

Comparing pool on population? Yes/No

N° of dropouts reported? Yes/No *=> Dropouts after randomization*

Reason dropouts? Reason dropouts

Comparing Drop-outs? Yes/No

*Are the individuals selected to participate in the study likely to be representative of the target

population? Very Likely/Somewhat Likely/Not Likely/Can’t tell

*(Q1) Participants are more likely to be representative of the target population if they are randomly selected from a comprehensive list of individuals in the target population (score very likely). They may not be representative if they are referred from a source (e.g. clinic) in a systematic manner (score somewhat likely) or self-referred (score not likely).*

*What percentage of selected individuals agreed to participate?

80-100% agreement/60-79%agreement/Less than 60% agreement/N.A./Can’t tell

*(Q2) Refers to the % of subjects in the control and intervention groups that agreed to participate in the study before they were assigned to intervention or control groups.*

1. **QUALITY**

**Intervention Quality**

Relevance and stakeholders acceptance? Yes/No

*=> Yes if something was mentioned how the intervention was received by participants and staff and/or something was mentioned why participants declined participating the intervention. If so, ad information in the box ‘barriers and facilitators’.*

Feasibility and discrepancy with the usual care? Yes/No

*=> Only yes if something was mentioned otherwise ‘No’*

Extent of Performance? Yes/No

*=> Attendance rate, reported difficulties…*

Barriers/facilitators Barriers and facilitators to participate

*Consistency of the intervention Yes/No/Can’t tell

*=>The number of participants receiving the intended intervention should be noted (consider both frequency and intensity). For example, the authors may have reported that at least 80 percent of the participants received the complete intervention. The authors should describe a method of measuring if the intervention was provided to all participants the same way. As well, the authors should indicate if subjects received an unintended intervention that may have influenced the outcomes. For example, co-intervention occurs when the study group receives an additional intervention (other than that intended). In this case, it is possible that the effect of the intervention may be over-estimated. Contamination refers to situations where the control group accidentally receives the study intervention. This could result in an under-estimation of the impact of the intervention.*

*Was the consistency of the intervention measured? Yes/No/Can’t tell

Who performed the intervention? Qualified Professional/Trained Professional/Not Qualified/Unknown/N.A.

*Is it likely that subjects received an unintended intervention (contamination or co-intervention) that may influence the results? Yes/No/Can’t tell

**Sampling Quality**

**Recruitment Homes**

How many approached and how many agreed? N° of NH that were contact/agreed to participate

Eligibility Criteria? Describe eligibility criteria

**Recruitment Participants**

Description? Description of participants characteristics

How and how many approached? Procedure of recruitment

How many agreed? + % N° (%) of participants that agreed

Eligibility and exclusion? Inclusion/exclusion criteria

*What percentage of participants received the allocated intervention or exposure of interest?

80-100% - 60-79% - less than 60% - Can’t tell

*=> % of the total participants that were allocated to the intervention group, allocation ratio*

Indicate the unit of allocation? Community – Institution – Practice/office – Individual

Indicate the unit of analysis? Community – Institution – Practice/office – Individual

*Unit or allocation/analysis: Institution = nursing home; Unit/ward/floor = practice/office*

1. **SUBGROUP**

**Depression** Instrument depression Baseline mean score

**Mental State** Instrument cognitive status Baseline mean score

**Physical Health** Instrument physical health Baseline mean score

*Give the main instrument used. Give the mean score of all participants (intervention + control group) based on baseline scores. If not available, use the baseline score of the intervention group. If also not available, use term like ‘cognitive impairment’, ‘bedridden’… mentioned by the author.*

1. **RESULTS**

**Results**

Description? Brief description of the results

Intervention Superior? Yes/No/Can’t tell

*Between group differences: only ‘yes’ if the outcome in the intervention group was significant compared to the control group; If only within group is mentioned: ‘can’t tell’*

**Participants**

**Intervention group**

How many analysed: N° participants in analysis

Drop-outs: N° dropouts / N° allocated

Mean pre: mean depression score / post: mean depression score

SD pre: SD score / post: SD score

*If not possible: SE, CI (95%)*

p-value (within-group) Post treatment

*If possible, not the adjusted mean score in order to make comparisons between the different studies!*

**Control Group**

How many participated: N° participants in analysis

Drop-outs: N° dropouts / N° allocated

Mean pre: mean depression score / post: mean depression score

SD pre: SD score / post: SD score

p-value (within-group) Post treatment

*Were there important differences between groups prior to the intervention? Yes/No/Can’t tell

*If yes, indicate percentage: 80-100% - 60-79% - less than 60% - Can’t tell

*By definition, a confounder is a variable that is associated with the intervention or exposure and causally related to the outcome of interest. Even in a robust study design, groups may not be balanced with respect to important variables prior to the intervention. The authors should indicate if confounders were controlled in the design (by stratification or matching) or in the analysis. Examples: Race, sex, age, marital status, Age, health status, baseline score on outcome measures.*

*If the allocation to intervention and control groups is randomized, the authors must report that the groups were balanced at baseline with respect to confounders (either in the text or a table, at least for one demographic!). If nothing is mentioned (either in text or table), score ‘weak’.*

*If no: ‘NA’; If yes, indicate the percentage of relevant confounders that were controlled either in design (e.g. stratification, matching) or analysis)? Design: In case of RCT score ‘Most -80 -100%’; In case of cluster and/or matched RCT: check whether this was controlled (either in design or analysis); if nothing is mentioned score ‘can’t tell’.*

*Were withdrawals and drop-outs reported in terms of numbers and/or reasons per group?

Yes/No/Can’t tell

***YES*** *if the authors describe BOTH the numbers and reasons for withdrawals and drop-outs.*

***NO*** *if either the numbers or reasons for withdrawals and drop-outs are not reported.*

*Indicate the percentage of participants completing the study. (If the percentage differs by groups, record the lowest). 80-100% - 60-79% - less than 60% - Can’t tell

*The percentage of participants completing the study refers to the % of subjects remaining in the study at the final data collection period in all groups (i.e. control and intervention groups).*

1. **EPHPP**

**GLOBAL RATING**

STRONG (no WEAK ratings) – 2 MODERATE (one WEAK rating) – 3 WEAK (two or more WEAK ratings)

**SELECTION BIAS**

**Strong:** The selected individuals are very likely to be representative of the target population (Q1 is 1)

**and** there is greater than 80% participation (Q2 is 1).

**Moderate:** The selected individuals are at least somewhat likely to be representative of the target

population (Q1 is 1 or 2); **and** there is 60 – 79% participation (Q2 is 2). ‘Moderate’ may also be assigned if Q1 is 1 or 2 and Q2 is 5 (can’t tell).

**Weak:** The selected individuals are not likely to be representative of the target population (Q1 is 3);

**or** there is less than 60% participation (Q2 is 3) **or** selection is not described (Q1 is 4); and the level of participation is not described (Q2 is 5).

**DESIGN**

**Strong:** will be assigned to those articles that described RCTs and CCTs.

**Moderate:** will be assigned to those that described a cohort analytic study, a case control study, a

cohort design, or an interrupted time series.

**Weak:** will be assigned to those that used any other method or did not state the method used.

**CONFOUNDERS**

**Strong:** will be assigned to those articles that controlled for at least 80% of relevant confounders (Q1

is 2); **or** (Q2 is 1).

**Moderate:** will be given to those studies that controlled for 60 – 79% of relevant confounders (Q1 is

1) **and** (Q2 is 2).

**Weak:** will be assigned when less than 60% of relevant confounders were controlled (Q1 is 1) **and**

(Q2 is 3) **or** control of confounders was not described (Q1 is 3) **and** (Q2 is 4).

**BLINDING**

**Strong:** The outcome assessor is not aware of the intervention status of participants (Q1 is 2); **and**

**t**he study participants are not aware of the research question (Q2 is 2).

**Moderate:** The outcome assessor is not aware of the intervention status of participants (Q1 is 2); **or**

the study participants are not aware of the research question (Q2 is 2); **or b**linding is not described (Q1 is 3 and Q2 is 3).

**Weak:** The outcome assessor is aware of the intervention status of participants (Q1 is 1); **and** the

study participants are aware of the research question (Q2 is 1).

**DATA COLLECTION METHODS**

**Strong:** The data collection tools have been shown to be valid (Q1 is 1); **and** the data collection tools

have been shown to be reliable (Q2 is 1).

**Moderate:** The data collection tools have been shown to be valid (Q1 is 1); **and** the data collection

tools have not been shown to be reliable (Q2 is 2) **or** reliability is not described (Q2 is 3).

**Weak:** The data collection tools have not been shown to be valid (Q1 is 2) **or** both reliability and

validity are not described (Q1 is 3 and Q2 is 3).

**WITHDRAWALS AND DROP-OUTS – a rating of:**

**Strong:** will be assigned when the follow-up rate is 80% or greater (Q2 is 1).

**Moderate:** will be assigned when the follow-up rate is 60 – 79% (Q2 is 2) **OR** Q2 is 5 (N/A).

**Weak:** will be assigned when a follow-up rate is less than 60% (Q2 is 3) or if the withdrawals and

drop-outs were not described (Q2 is 4).

##
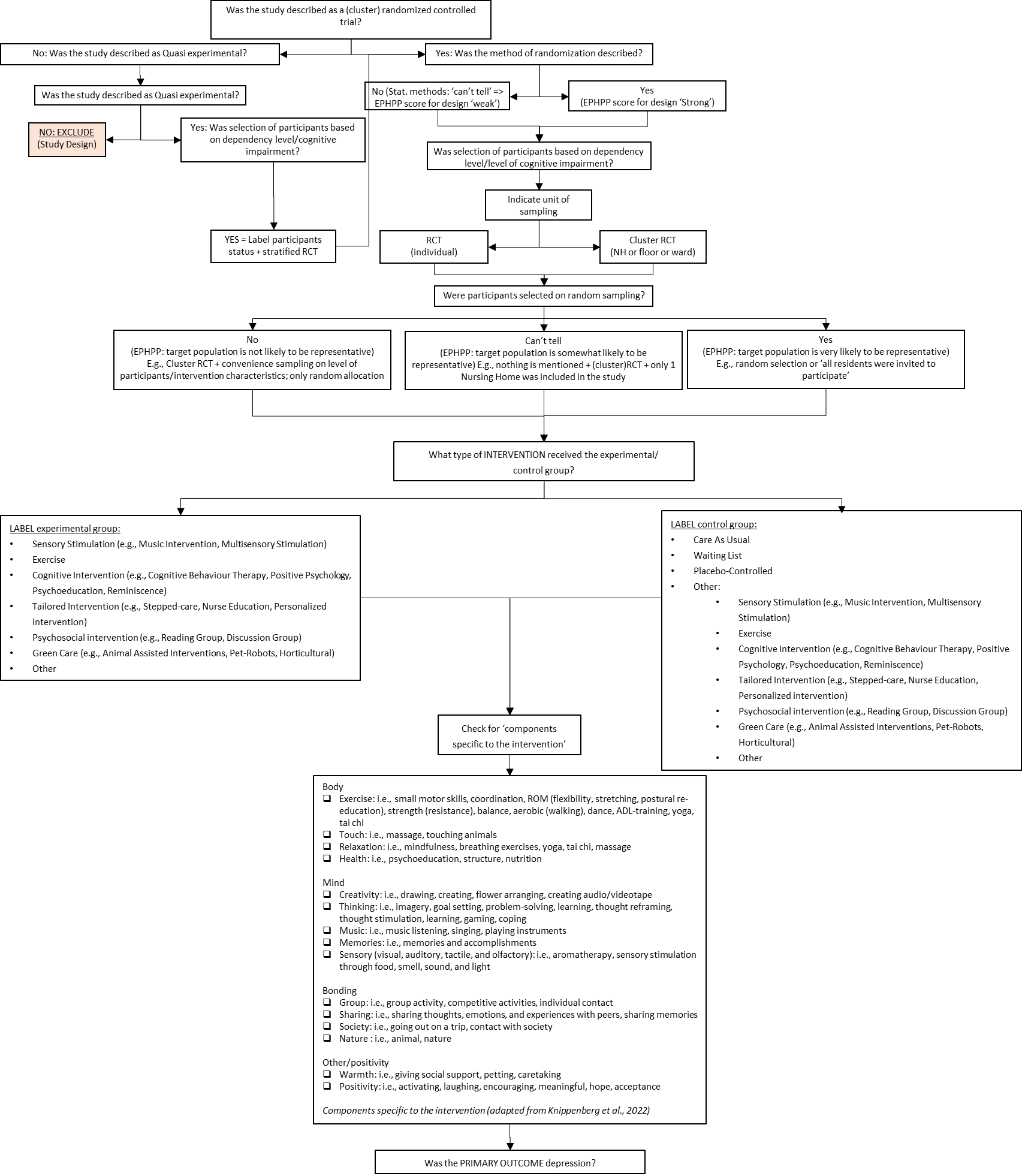
Figure 2.1. Decision tree


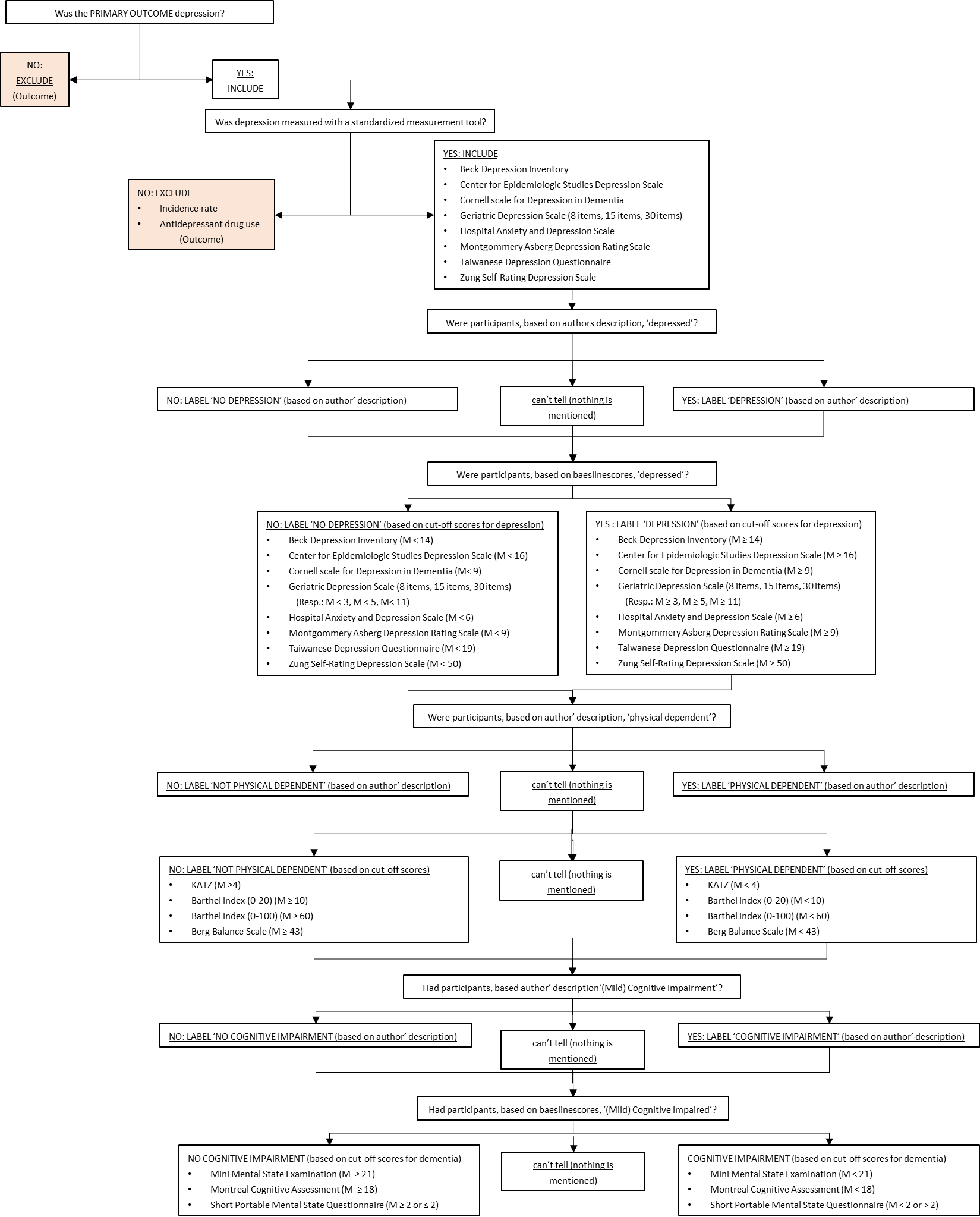

Supplement: Supplementary file 2 — Supplementary Material 2. [file 12877_2024_5117_MOESM2_ESM.docx]
