# Supplementary material for: A bayesian network meta-analysis to explore modifying factors in randomized controlled trials: what works for whom to reduce depression in nursing home residents?
Source: BMC Geriatr. 2024 Jun 14;24:518. doi: 10.1186/s12877-024-05117-8 (PMC11177425; doi:10.1186/s12877-024-05117-8)
Supplement: Supplementary file 3 — Supplementary Material 3. [file 12877_2024_5117_MOESM3_ESM.docx]

# Additional File 3 – Quality Assessment

###

This appendix contains information with respect to the bias and certainty assessment. First, a brief overview of the methodology is provided. Second, results of the different assessments are summarized in tables, and additional funnel plots are provided.

# Quality Assessment (Meader, 2014; Puhan et al., 2014; Salanti et al., 2014)

The quality of evidence from the network meta-analysis (GADE assessment) was based on recommendations of Meader (2014), Puhan et al. (2014) and Salanti et al. (2014). We used the checklist of Meader (2014) as outset for the GRADE assessment.

## Study limitations (EPHPP)

To assess confidence in the included studies, ratings of the EPHPP tool were used. Table 1 provides an overview of the EPHPP questions that were used to assess the potential study limitations as reported in the checklist of Meader (2014).

| Selection Bias  *Are the individuals selected to participate in the study likely to be representative of the target population?*  *(EPHPP score A – Selection Bias)* | NO Bias (Green): Target population is very likely to be representative. There were randomly selected, or all residents in the participating NH were invited.  SOME Bias (Yellow): Target population is somewhat likely to be representative. Authors did not mention selection procedure, but study was described as RCT.  Selection Bias (Red) : Target population is not likely to be representative. Authors did not describe the level of participation, or convenience sampling was used on participants level or intervention characteristics (e.g., not being afraid of dogs). |
| --- | --- |
| Allocation Bias  *Was the study described as RCT? Was the method of randomization described?*  *Was the method appropriate? (EPHPP score B – Design)* | NO Bias (Green): Study was described as RCT. Authors did describe the method of randomization. This method was appropriate.  Some Bias (Yellow): Study was not described as RCT (nothing mentioned or quasi-experimental). Authors did describe the method of randomization. This method was appropriate.  Allocation Bias (Red): Study was described as RCT, but authors did not describe method of randomization. |
| Performance Bias  *Were participants blinded to the research question?*  *(EPHPP score D – Blinding)* | NO Bias (Green): The study  participants are not aware of the research question.  Some Bias (Yellow): Blinding is not described.  Detection Bias (Red): The study participants are aware of the research question. |
| Detection Bias  *Was allocation status of the participants blinded to the outcome assessors?*  *(EPHPP score D – Blinding)* | NO Bias (Green): The outcome assessor is not aware of the intervention status of participants.  Some Bias (Yellow): Blinding is not described.  Detection Bias (Red): The outcome assessor is aware of the intervention status of participants. |
| Was an objective outcome used?  *(EPHPP score F - Data collection methods)* | All data collection tools were standardized, since this was one of our inclusion criteria. Therefore we did not expect any subjectiveness in outcome measurements. |
| Reporting Bias  *(% completers + Intention to treat analysis)* | NO Bias (Green): 80% of the selected participants completed the intervention and/or analysis were based on intention to treat principles.  Some Bias (Yellow): 1) 60-79% of the selected participants completed the intervention, and analysis was based on intention to treat principles, or 2) drop-outs was not mentioned, and analysis was based on intention to treat principles.  Reporting Bias (Red); 1) less than 60% of the participants completed the intervention; 2) 60-79% of the selected participants completed the intervention or % completers was not mentioned, and analysis was not based on intention to treat principles. |

## Inconsistency of the network

Inconsistency of the network was rated based on joint consideration of statistical heterogeneity (measured by *I²*), and statistical inconsistency (direct versus indirect evidence) of the different comparison arms (Salanti et al., 2014). Table 2 provides an overview of the criteria and questions that were used to assess both, i.e., statistical heterogeneity and inconsistency (Meader, 2014).

| Statistical inconsistency (Direct versus indirect evidence in the network, based on the note splitting method and exploring confidence intervals in the forest plot) | |
| --- | --- |
| Did point estimates vary widely?  *A difference of .30 MD between direct and indirect effect was considered as threshold.* | No (green)  Yes (Red) |
| To what extent did confidence interval overlap?  *Recommendations of the checklist of Meader (2014) were used. In addition, Bayesian p-value was evaluated; a threshold of p<0.05 was considered as a statistical significant difference between direct and indirect evidence.* | Substantial overlap (Green): all confidence intervals (i.e., direct versus indirect evidence of the network) overlap with the confidence interval of the network.  Some overlap (Yellow): confidence intervals overlap but not both of them (direct and indirect) overlap with the confidence interval of the network.  No overlap (Red): Confidence intervals of both direct and indirect effects do not overlap with the confidence interval of the network. |
| Was the direction of the effect consistent?  *To evaluate if the direction of the effect was consistent, the direction of the MD (Mean Difference) of both direct and indirect effect was compared.* | Yes, the direction of both means is consistent(Green)  No (Red) |
| Statistical heterogeneity (measure by *I²* for SMD (Hedges’ *g*) - see forest plot) | |
| What was the magnitude of statistical heterogeneity in the comparison arms? | Low (*I²* <40%) (Green)  Moderate (*I²* 40-60%) (Yellow)  High (*I²* >60%) (Red) |
| Was the test for heterogeneity statistically significant (<0.05)? | Not statistical significant (≥0.05) (Green)  Statistical significant (<0.05) (Red) |

## Indirectness

Indirectness of the network was rated based on recommendations of Meader (2014). The inclusion criteria of this research were used to evaluate the applicable questions.

| Were the populations in included studies applicable to the decision context? | Highly applicable (Green)  Applicable (Yellow)  Poorly applicable (Red) |
| --- | --- |
| Were the interventions in the included studies applicable to the decision context? | Highly applicable (Green)  Applicable (Yellow)  Poorly applicable (Red) |
| Was the included outcome not a surrogate outcome? | Yes, not a surrogate (Green)  No (Red) |
| Was the outcome timeframe sufficient? | Sufficient (trials between 4 and 16 weeks) (Green)  Insufficient (trials <4 weeks and >16 weeks) (Red) |

## Imprecision

Imprecision of the network was rated by examining the 95% confidence intervals of the different comparison arms (Salanti et al., 2014). Clinically relevant effect sizes, i.e., 95% confidence intervals of the different comparison arms based on both direct and indirect evidence, were further evaluated with respect to the potential benefit and harms (Salanti et al., 2014).

| Was the confidence interval for the pooled estimate consistent with no harm? | Yes – 1) 95% CI included zero or 2) the 95% CI did not include zero and the intervention was beneficial for the participants (Green)  No - ) 95% CI did not include zero and the intervention was harmful for the participants (Red) |
| --- | --- |
| What is the magnitude of the median sample size of the studies n the different comparison arms? | High (i.e., 300 participants) (Green)  Intermediate (i.e., 100-300 participants) (Yellow)  Low (i.e., <100 participants) (Red) |
| What was the magnitude of the number of included studies? | Large (e.g. >10 studies) (Green)  Moderate (e.g. 5-10 studies) (Yellow)  Small (e.g. <5 studies) (Red) |
| Was there no evidence of serious harm associated with treatment? | No (Green)  Yes (Red) |

## Publication Bias (other considerations)

| Did the authors conduct a comprehensive search? | Yes (Green)  No (Red) |
| --- | --- |
| Did the authors search for grey literature? | Yes (Green)  Not active (articles were not excluded; but trial registration and reference sections of other reviews were explored)  No (Red) |
| Authors did not apply restrictions to study selection on the basis of language? | Yes (Green)  No (Red) |
| There was no industry influence on studies included in the review? | Yes (Green)  No (Red) |
| There was no evidence of funnel plot asymmetry within the network? (the whole network was evaluated) | Yes (Green)  No (Red) |

# EPHPP assessment

##### **Table 3.1. EPHPP assessment of the included studies (N=111)**

|  | Global rating* | A rate* | B rate* | C rate* | D rate* | E rate* | F rate* |
| --- | --- | --- | --- | --- | --- | --- | --- |
| Abraham et al.,1992 | 1 | 1 | 1 | 3 | 2 | 2 | 1 |
| Bailey et al.,2017 | 1 | 1 | 1 | 3 | 2 | 3 | 1 |
| Badrasawi et al.,2013 | 2 | 2 | 3 | 3 | 1 | 2 | 3 |
| Bergh et al.,2012 | 2 | 2 | 3 | 3 | 3 | 2 | 1 |
| Chapman et al.,2007 | 1 | 1 | 3 | 3 | 2 | 2 | 1 |
| Chiang et al.,2010 | 3 | 2 | 3 | 3 | 2 | 3 | 2 |
| Cook, E. A.,1991 | 2 | 3 | 1 | 3 | 2 | 2 | 2 |
| Dowling et al.,2007 | 1 | 2 | 1 | 3 | 1 | 2 | 1 |
| Elias et al.,2020 | 1 | 2 | 1 | 3 | 1 | 3 | 3 |
| Goldwasser et al.,1987 | 2 | 2 | 1 | 3 | 2 | 2 | 3 |
| Hsieh et al.,2010 | 2 | 2 | 1 | 3 | 2 | 2 | 3 |
| Hashimoto et al.,2017 | 2 | 3 | 1 | 3 | 3 | 2 | 3 |
| Hsu et al.,2009 | 3 | 2 | 3 | 3 | 2 | 2 | 3 |
| Hyer et al.,2009 | 2 | 2 | 1 | 3 | 2 | 2 | 3 |
| Kennedy et al.,2000 | 2 | 1 | 3 | 3 | 3 | 3 | 3 |
| Kolberg et al.,2021 | 2 | 3 | 3 | 3 | 1 | 2 | 2 |
| Konnert et al.,2009 | 2 | 2 | 3 | 3 | 2 | 2 | 1 |
| Lan et al.,2019 | 2 | 1 | 3 | 3 | 2 | 2 | 3 |
| Lichtenberg et al.,2005 | 2 | 2 | 1 | 3 | 2 | 2 | 3 |
| Llewellyn-Jones et al.,1999 | 3 | 2 | 3 | 3 | 2 | 2 | 2 |
| Luijpen et al.,2018 | 1 | 2 | 1 | 3 | 2 | 2 | 1 |
| Lok et al.,2004 | 2 | 2 | 3 | 3 | 1 | 3 | 3 |
| Lopes et al.,2016 | 2 | 1 | 3 | 3 | 2 | 2 | 3 |
| Luo et al.,2020 | 1 | 1 | 1 | 3 | 1 | 2 | 2 |
| Moghadam et al.,2018 | 1 | 2 | 1 | 3 | 2 | 2 | 1 |
| Parola et al.,2016 | 3 | 3 | 3 | 3 | 2 | 2 | 3 |
| Onega et al.,2018 | 2 | 2 | 1 | 3 | 2 | 3 | 3 |
| Proctor et al.,1999 | 2 | 1 | 3 | 3 | 2 | 2 | 3 |
| Rondonelli et al.,2021 | 2 | 2 | 3 | 3 | 3 | 2 | 3 |
| Resnick et al.,2010 | 1 | 1 | 1 | 3 | 2 | 2 | 3 |
| Royer et al.,2012 | 1 | 1 | 3 | 3 | 2 | 2 | 1 |
| Stange et al.,2015 | 1 | 1 | 3 | 3 | 1 | 2 | 2 |
| Soniya, G.,2013 | 1 | 2 | 3 | 1 | 2 | 2 | 1 |
| Ulfvarson et al.,2003 | 2 | 1 | 3 | 3 | 2 | 3 | 2 |
| Van Bogaert et al.,2016 | 3 | 3 | 3 | 3 | 2 | 2 | 3 |
| van Dongen et al.,2000 | 2 | 1 | 3 | 3 | 3 | 2 | 3 |
| Wang et al.,2007 | 2 | 1 | 3 | 3 | 2 | 3 | 3 |
| Westerhof et al.,2018 | 1 | 1 | 3 | 3 | 1 | 3 | 2 |
| Yang et al.,2016 | 2 | 2 | 1 | 3 | 2 | 2 | 3 |
| Alidoost et al.,2021 | 2 | 1 | 3 | 3 | 2 | 3 | 3 |
| Aravich et al.,2021 | 1 | 1 | 1 | 3 | 2 | 2 | 1 |
| Aravich et al.,2021 | 1 | 1 | 1 | 3 | 2 | 2 | 1 |
| Bae et al.,2020 | 1 | 1 | 1 | 3 | 2 | 2 | 2 |
| Buettner et al.,2002 | 1 | 2 | 1 | 3 | 2 | 2 | 1 |
| Cancela et al.,2016 | 1 | 2 | 3 | 3 | 1 | 2 | 1 |
| Carroll et al.,1998 | 1 | 2 | 1 | 3 | 1 | 3 | 1 |
| Celko et al.,2014 | 2 | 3 | 1 | 3 | 2 | 2 | 3 |
| Chen et al.,2017 | 2 | 1 | 3 | 3 | 2 | 3 | 3 |
| Chen et al.,2015 | 2 | 1 | 3 | 3 | 2 | 3 | 3 |
| Chen et al.,2021 | 3 | 2 | 3 | 3 | 2 | 2 | 3 |
| Cheng et al.,2011 | 1 | 2 | 1 | 3 | 1 | 2 | 1 |
| Hsu et al.,2016 | 1 | 1 | 3 | 3 | 1 | 3 | 3 |
| Chiang et al.,2019 | 2 | 2 | 1 | 3 | 2 | 2 | 3 |
| Chin et al.,2004 | 2 | 3 | 3 | 3 | 2 | 2 | 1 |
| Ching-Teng, et al.,2019 | 1 | 1 | 1 | 3 | 2 | 2 | 3 |
| Chiu et al.,2019 | 2 | 2 | 1 | 3 | 2 | 2 | 3 |
| Colombo et al.,2005 | 1 | 3 | 1 | 3 | 2 | 2 | 1 |
| Conradson et al.,2010 | 3 | 2 | 3 | 3 | 2 | 2 | 3 |
| Cooke et al.,2010 | 3 | 3 | 3 | 3 | 2 | 3 | 3 |
| Cordes et al.,2021 | 2 | 1 | 3 | 3 | 2 | 2 | 2 |
| Davison et al.,2017 | 1 | 1 | 3 | 2 | 1 | 3 | 2 |
| Davison et al.,2016 | 2 | 1 | 3 | 3 | 2 | 3 | 2 |
| Dozeman et al.,2011 | 1 | 1 | 3 | 3 | 2 | 2 | 1 |
| Eggermont et al.,2009 | 1 | 1 | 1 | 3 | 2 | 3 | 2 |
| Fakhari et al.,2017 | 2 | 2 | 3 | 3 | 1 | 2 | 3 |
| Friedman et al.,2015 | 1 | 1 | 3 | 3 | 1 | 2 | 3 |
| Ugur et al.,2016 | 3 | 2 | 3 | 3 | 2 | 3 | 3 |
| Alka et al.,2021 | 3 | 2 | 3 | 3 | 2 | 2 | 3 |
| Hsu et al.,2019 | 1 | 1 | 3 | 1 | 2 | 3 | 3 |
| Hutson et al.,2014 | 1 | 1 | 3 | 1 | 2 | 2 | 3 |
| Joranson et al.,2015 | 1 | 1 | 1 | 3 | 1 | 3 | 3 |
| Krishnamurthy et al.,2007 | 3 | 2 | 3 | 3 | 2 | 2 | 2 |
| Le roux et al.,2009 | 1 | 1 | 1 | 3 | 2 | 3 | 3 |
| Lee et al.,2020 | 1 | 2 | 3 | 1 | 2 | 2 | 1 |
| Lin et al.,2019 | 1 | 1 | 1 | 3 | 2 | 2 | 3 |
| Lok et al.,2017 | 2 | 2 | 3 | 3 | 1 | 2 | 3 |
| Low et al.,2012 | 2 | 2 | 3 | 3 | 1 | 2 | 3 |
| McCurren et al.,1999 | 2 | 2 | 1 | 3 | 2 | 2 | 2 |
| McSweeney et al.,2012 | 2 | 1 | 3 | 3 | 2 | 2 | 3 |
| Moghaddasifar et al.,2018 | 1 | 2 | 1 | 3 | 2 | 2 | 1 |
| Moyle et al.,2013 | 2 | 2 | 3 | 3 | 2 | 2 | 1 |
| Olsen et al.,2016 | 1 | 1 | 3 | 3 | 1 | 2 | 3 |
| Parra et al.,2021 | 1 | 1 | 1 | 3 | 2 | 3 | 3 |
| Pérez-Ros et al.,2019 | 2 | 2 | 3 | 3 | 2 | 2 | 3 |
| Robinson et al.,2013 | 2 | 1 | 3 | 3 | 2 | 3 | 3 |
| Rodriguez-Mansilla et al.,2015 | 2 | 2 | 3 | 1 | 3 | 2 | 3 |
| Scilley et al.,2007 | 1 | 3 | 1 | 3 | 1 | 2 | 3 |
| Siregar et al.,2019 | 1 | 1 | 3 | 3 | 1 | 2 | 1 |
| Sollami et al.,2017 | 1 | 2 | 1 | 1 | 2 | 3 | 1 |
| Tapps et al.,2013 | 1 | 1 | 1 | 1 | 2 | 2 | 2 |
| Thodberg et al.,2016 | 2 | 2 | 1 | 3 | 2 | 2 | 3 |
| Todri et al.,2019 | 3 | 2 | 3 | 3 | 2 | 2 | 3 |
| Tsai et al.,2011 | 1 | 1 | 1 | 3 | 2 | 3 | 1 |
| Tsai et al.,2008 | 2 | 2 | 3 | 3 | 2 | 2 | 1 |
| Tse et al.,2017 | 2 | 2 | 3 | 3 | 2 | 2 | 1 |
| Tseng et al.,2006 | 3 | 2 | 3 | 3 | 2 | 2 | 2 |
| Teng et al.,2021 | 3 | 3 | 3 | 3 | 2 | 2 | 3 |
| Underwood et al.,2013 | 1 | 1 | 3 | 3 | 1 | 2 | 1 |
| Wang et al.,2011 | 3 | 2 | 3 | 3 | 2 | 3 | 3 |
| Williams et al.,2008 | 2 | 1 | 3 | 3 | 2 | 2 | 2 |
| Wilson et al.,2010 | 1 | 1 | 1 | 3 | 2 | 2 | 3 |
| Meléndez-Moral et al.,2013 | 1 | 1 | 1 | 3 | 2 | 2 | 1 |
| Ambrosi et al.,2019 | 2 | 1 | 3 | 3 | 2 | 2 | 3 |
| Travers et al.,2013 | 2 | 1 | 3 | 3 | 2 | 2 | 2 |
| Lutwack-Bloom et al.,2008 | 2 | 2 | 3 | 3 | 3 | 2 | 1 |
| Petersen et al.,2017 | 1 | 1 | 3 | 3 | 1 | 2 | 1 |
| Elsegood et al.,2012 | 1 | 1 | 1 | 3 | 2 | 2 | 3 |
| Hamzehzadeh et al.,2018 | 1 | 1 | 1 | 3 | 2 | 2 | 2 |
| Biasutti,2021 | 2 | 1 | 3 | 3 | 2 | 2 | 3 |
| Baker et al.,2022 | 2 | 3 | 3 | 2 | 2 | 2 | 1 |
| Costa et al.,2018 | 1 | 2 | 1 | 3 | 1 | 2 | 2 |

**Note: 1=weak; 2=moderate; 3=strong*

# GRADE assessment

##### **Table 3.2. GRADE assessment of the network (N=111)**

| treat1 | treat2 |  | Selection Bias | Allocation bias | Detection Bias | Performance Bias | Instrument tools validated? | Reporting bias | Study Limitations | Point estimations widely? | Confidence Interval overlap? | Direction of the effect consistent? | Heterogeinity | Heterogeinity sigificant? | Inconsistency | Population applicable? | Interventions applicable? | Surrogate outcome? | Sufficient timeframe? | Direct comparisons? | Indirectness | Benefit and hamr? | Median sample size? | Number of included studies? | Serious harm? | Imprecision | Comprehensive search? | Grey literature? | Restrictions based on language? | Industry influence ? | Funnel Plot assymetrie? | Discrepanty unpublished trials? | Publication bias |
| --- | --- | --- | --- | --- | --- | --- | --- | --- | --- | --- | --- | --- | --- | --- | --- | --- | --- | --- | --- | --- | --- | --- | --- | --- | --- | --- | --- | --- | --- | --- | --- | --- | --- |
| CI | **PS** |  | 1 | 2 | 3 | 1 | 3 | 2 | 2 | 3 | 3 | 3 | 3 | 3 | 3 | 3 | 2 | 3 | 2 | 1 | 2 | 3 | 1 | 2 | 3 | 2 | 3 | 2 | 3 | 3 | 1 | 2 | 2 |
| CI | **Interv Plac** |  | 2 | 1 | 1 | 1 | 3 | 3 | 2 | 3 | 3 | 3 |  |  | 3 | 3 | 2 | 3 | 3 | 1 | 2 | 3 | 1 | 1 | 3 | 2 | 3 | 2 | 3 | 3 | 1 | 2 | 2 |
| CI | **CAU** |  | 2 | 1 | 2 | 1 | 3 | 2 | 2 | 3 | 3 | 3 | 1 | 1 | 2 | 3 | 2 | 3 | 3 | 1 | 2 | 3 | 1 | 3 | 3 | 3 | 3 | 2 | 3 | 3 | 1 | 2 | 2 |
| CI | **WL** |  | 1 | 2 | 1 | 2 | 3 | 2 | 2 | 1 | 3 | 3 | 1 | 1 | 2 | 3 | 2 | 3 | 3 | 1 | 2 | 3 | 1 | 1 | 3 | 2 | 3 | 2 | 3 | 3 | 1 | 2 | 2 |
| CI | **RT** |  | 2 | 1 | 2 | 1 | 3 | 3 | 2 | 1 | 3 | 3 |  |  | 2 | 3 | 2 | 3 | 3 | 1 | 2 | 3 | 1 | 1 | 3 | 2 | 3 | 2 | 3 | 3 | 1 | 2 | 2 |
| TI | **CAU** |  | 1 | 3 | 3 | 2 | 3 | 3 | 3 | 3 | 3 | 3 | 1 | 1 | 2 | 3 | 2 | 3 | 2 | 1 | 2 | 3 | 1 | 2 | 3 | 2 | 3 | 2 | 3 | 3 | 1 | 2 | 2 |
| TI | **WL** |  | 1 | 3 | 2 | 1 | 3 | 1 | 2 | 3 | 3 | 1 | 1 | 1 | 2 | 3 | 2 | 3 | 3 | 1 | 2 | 3 | 2 | 1 | 3 | 2 | 3 | 2 | 3 | 3 | 1 | 2 | 2 |
| PS | **CAU** |  | 2 | 2 | 2 | 1 | 3 | 2 | 2 | 1 | 3 | 3 | 1 | 1 | 2 | 3 | 2 | 3 | 3 | 1 | 2 | 3 | 1 | 2 | 3 | 2 | 3 | 2 | 3 | 3 | 1 | 2 | 2 |
| PS | **Interv Plac** |  | 2 | 3 | 2 | 2 | 3 | 1 | 2 | 3 | 3 | 3 |  |  | 3 | 3 | 2 | 3 | 3 | 1 | 2 | 3 | 1 | 1 | 3 | 2 | 3 | 2 | 3 | 3 | 1 | 2 | 2 |
| SS | **CAU** |  | 2 | 2 | 3 | 1 | 3 | 2 | 2 | 3 | 3 | 3 | 1 | 1 | 2 | 3 | 2 | 3 | 3 | 1 | 2 | 3 | 1 | 3 | 3 | 3 | 3 | 2 | 3 | 3 | 1 | 2 | 2 |
| SS | **Interv Plac** |  | 2 | 2 | 1 | 3 | 3 | 2 | 2 | 3 | 3 | 3 | 1 | 3 | 3 | 3 | 2 | 3 | 1 | 1 | 2 | 3 | 1 | 1 | 3 | 2 | 3 | 2 | 3 | 3 | 1 | 2 | 2 |
| SS | **Ex** |  | 1 | 3 | 3 | 1 | 3 | 3 | 2 | 1 | 3 | 1 |  |  | 2 | 3 | 2 | 3 | 3 | 1 | 2 | 3 | 1 | 1 | 3 | 2 | 3 | 2 | 3 | 3 | 1 | 2 | 2 |
| SS | **PS** |  | 2 | 3 | 3 | 2 | 3 | 2 | 3 | 3 | 3 | 3 | 2 | 3 | 3 | 3 | 2 | 3 | 3 | 1 | 2 | 3 | 1 | 1 | 3 | 2 | 3 | 2 | 3 | 3 | 1 | 2 | 2 |
| SS | **RT** |  | 2 | 1 | 2 | 1 | 3 | 3 | 2 | 1 | 3 | 3 |  |  | 2 | 3 | 2 | 3 | 3 | 1 | 2 | 3 | 1 | 1 | 3 | 2 | 3 | 2 | 3 | 3 | 1 | 2 | 2 |
| GC | **CAU** |  | 2 | 1 | 2 | 1 | 3 | 2 | 2 | 3 | 3 | 3 | 2 | 3 | 3 | 3 | 2 | 3 | 3 | 1 | 2 | 3 | 1 | 2 | 3 | 2 | 3 | 2 | 3 | 3 | 1 | 2 | 2 |
| GC | **RT** |  | 1 | 3 | 1 | 1 | 3 | 3 | 2 | 3 | 3 | 1 |  |  | 2 | 3 | 2 | 3 | 3 | 1 | 2 | 3 | 1 | 1 | 3 | 2 | 3 | 2 | 3 | 3 | 1 | 2 | 2 |
| GC | **PS** |  | 2 | 2 | 3 | 1 | 3 | 1 | 2 | 3 | 3 | 1 | 1 | 1 | 2 | 3 | 2 | 3 | 2 | 1 | 2 | 3 | 1 | 1 | 3 | 2 | 3 | 2 | 3 | 3 | 1 | 2 | 2 |
| Neuro | **CAU** |  | 2 | 2 | 1 | 1 | 3 | 2 | 2 | 1 | 3 | 1 | 1 | 1 | 1 | 3 | 2 | 3 | 3 | 1 | 2 | 3 | 1 | 1 | 3 | 2 | 3 | 2 | 3 | 3 | 1 | 2 | 2 |
| Neuro | **Placebo** |  | 1 | 3 | 3 | 2 | 3 | 2 | 2 | 3 | 3 | 3 | 1 | 1 | 2 | 3 | 2 | 3 | 2 | 1 | 2 | 3 | 1 | 3 | 3 | 3 | 3 | 2 | 3 | 3 | 1 | 2 | 2 |
| Neuro | **WL** |  | 3 | 1 | 1 | 1 | 3 | 3 | 2 | 1 | 3 | 1 |  |  | 2 | 3 | 2 | 3 | 3 | 1 | 2 | 3 | 2 | 1 | 3 | 2 | 3 | 2 | 3 | 3 | 1 | 2 | 2 |
| RT | **CAU** |  | 2 | 2 | 2 | 1 | 3 | 3 | 2 | 3 | 3 | 3 | 1 | 1 | 2 | 3 | 2 | 3 | 3 | 1 | 2 | 3 | 1 | 3 | 3 | 3 | 3 | 2 | 3 | 3 | 1 | 2 | 2 |
| RT | **PS** |  | 1 | 2 | 1 | 1 | 3 | 2 | 2 | 1 | 3 | 1 | 3 | 3 | 2 | 3 | 2 | 3 | 3 | 1 | 2 | 3 | 1 | 1 | 3 | 2 | 3 | 2 | 3 | 3 | 1 | 2 | 2 |
| RT | **WL** |  | 2 | 3 | 2 | 1 | 3 | 1 | 2 | 1 | 3 | 3 |  |  | 2 | 3 | 2 | 3 | 3 | 1 | 2 | 3 | 2 | 1 | 3 | 2 | 3 | 2 | 3 | 3 | 1 | 2 | 2 |
| Ex | **CAU** |  | 2 | 3 | 2 | 1 | 3 | 2 | 2 | 1 | 3 | 3 | 1 | 1 | 2 | 3 | 2 | 3 | 2 | 1 | 2 | 3 | 1 | 3 | 3 | 3 | 3 | 2 | 3 | 3 | 1 | 2 | 2 |
| Ex | **WL** |  | 2 | 3 | 3 | 1 | 3 | 2 | 2 | 1 | 3 | 3 | 1 | 1 | 2 | 3 | 2 | 3 | 2 | 1 | 2 | 3 | 1 | 1 | 3 | 2 | 3 | 2 | 3 | 3 | 1 | 2 | 2 |
| Ex | **CI** |  | 2 | 3 | 3 | 1 | 3 | 2 | 2 | 3 | 3 | 1 | 1 | 3 | 2 | 3 | 2 | 3 | 3 | 1 | 2 | 3 | 1 | 1 | 3 | 2 | 3 | 2 | 3 | 3 | 1 | 2 | 2 |
| Ex | **Interv Plac** |  | 2 | 1 | 1 | 1 | 3 | 1 | 1 | 1 | 3 | 3 |  |  | 2 | 3 | 2 | 3 | 3 | 1 | 2 | 3 | 1 | 1 | 3 | 2 | 3 | 2 | 3 | 3 | 1 | 2 | 2 |
| Ex | **PS** |  | 2 | 3 | 3 | 1 | 3 | 2 | 2 | 1 | 3 | 1 | 3 | 3 | 2 | 3 | 2 | 3 | 2 | 1 | 2 | 3 | 1 | 2 | 3 | 2 | 3 | 2 | 3 | 3 | 1 | 2 | 2 |
| Ex | **TI** |  | 1 | 3 | 1 | 1 | 3 | 1 | 2 | 1 | 3 | 1 |  |  | 2 | 3 | 2 | 3 | 1 | 1 | 2 | 3 | 3 | 1 | 3 | 3 | 3 | 2 | 3 | 3 | 1 | 2 | 2 |
| Robots | **CAU** |  | 1 | 2 | 1 | 1 | 3 | 2 | 2 | 1 | 3 | 1 | 3 | 3 | 2 | 3 | 2 | 3 | 3 | 1 | 2 | 3 | 1 | 1 | 3 | 2 | 3 | 2 | 3 | 3 | 1 | 2 | 2 |
| Robots | **PS** |  | 2 | 2 | 2 | 1 | 3 | 2 | 2 | 1 | 3 | 3 | 3 | 3 | 3 | 3 | 2 | 3 | 3 | 1 | 2 | 3 | 1 | 1 | 3 | 2 | 3 | 2 | 3 | 3 | 1 | 2 | 2 |

*Notes (Table 2): CI = Cognitive Interventions; PS = Psychosocial Interventions; SS = Sensory Stimulation Interventions; RT = Reminiscence Therapy; CAU = CAU; Ex = Exercise Interventions; WL = Waiting List; TI = Tailored Interventions; GC = Green Care; Robots = Pet-Robots; IntervPlac = Placebo Intervention; Neuro = Neurobiological interventions; Placebo = neurobiological placebo’s; *Note: 1=weak (red); 2=moderate (yellow); 3=strong (green)*

##### **Figure 3.1. Funnel Plot (N=118)**

##### **
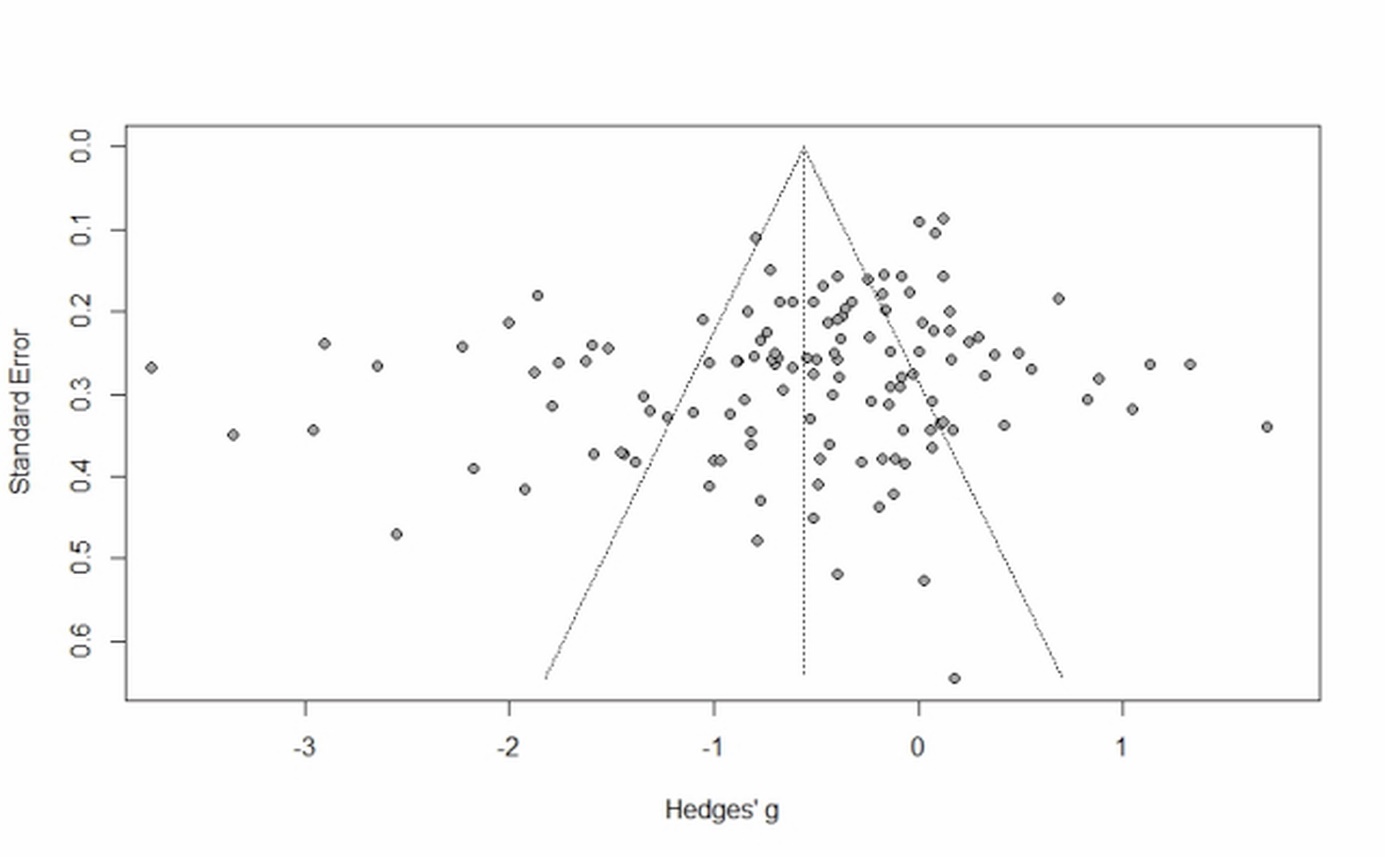
**

##### **Figure 3.2. Funnel Plot (N=111)**

**
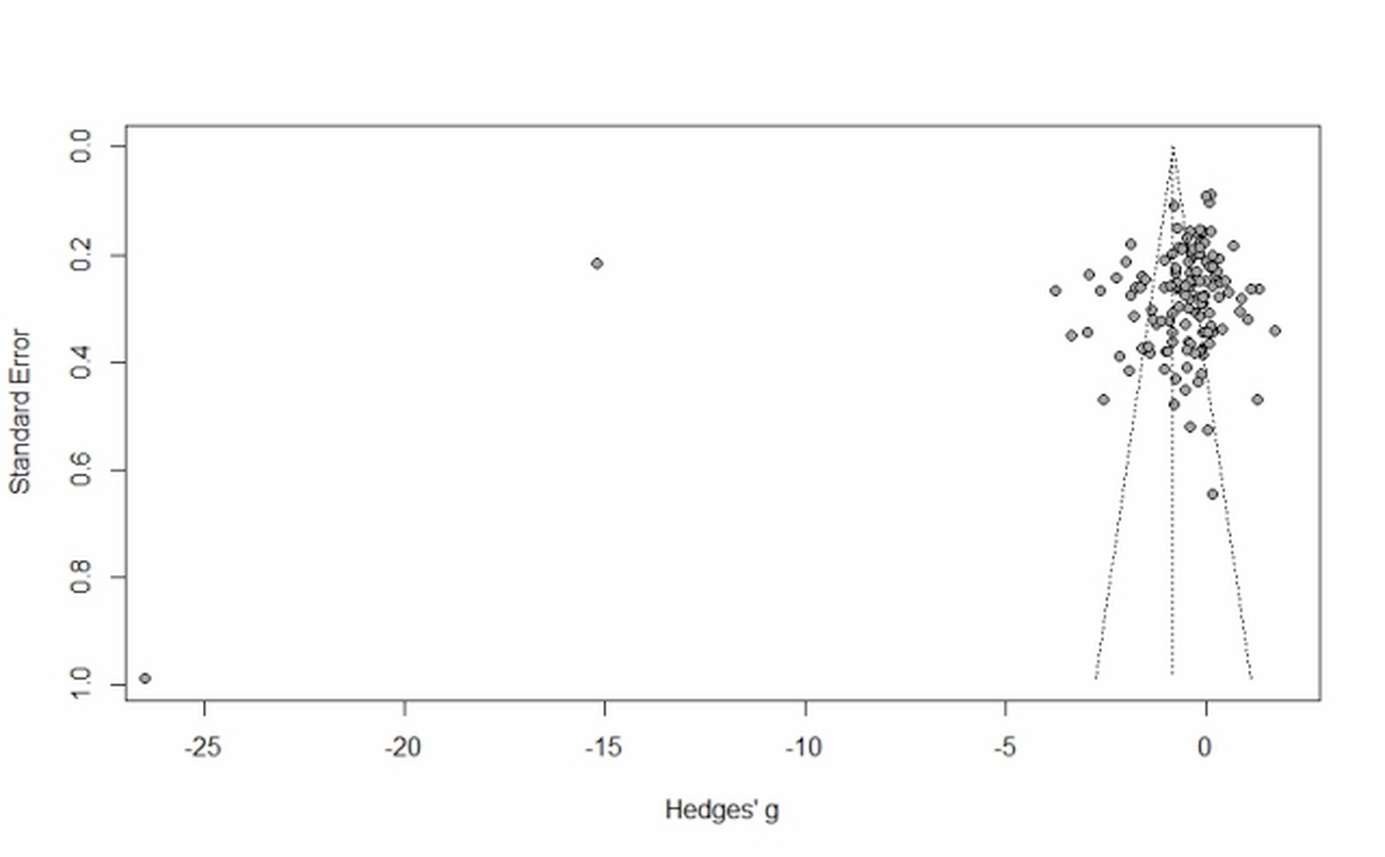
**
