# Supplementary material for: A bayesian network meta-analysis to explore modifying factors in randomized controlled trials: what works for whom to reduce depression in nursing home residents?
Source: BMC Geriatr. 2024 Jun 14;24:518. doi: 10.1186/s12877-024-05117-8 (PMC11177425; doi:10.1186/s12877-024-05117-8)
Supplement: Supplementary file 4 — Supplementary Material 4. [file 12877_2024_5117_MOESM4_ESM.docx]

**Additional File 4 - Study Characteristics**

In this appendix, an overview of the main characteristics of the included studies is provided.

**Table 4.1. Study characteristic**

| Author(s) | IG | N° IG | CG | N° CG | Sign.^A^ | Trial duration^b^ | Continent | Instrument Depression | (Mild) cognitive impairment | Dependent | Body | Mind | Bonding | Positivity |
| --- | --- | --- | --- | --- | --- | --- | --- | --- | --- | --- | --- | --- | --- | --- |
| Cognitive Interventions (N=17) | | | | | | | | | | | | | | |
| Abraham et al.,1992 | Cognitive Behavioral Therapy | 19 | Psychosocial (Discussion Group) | 8 | No | 20 | America | GDS-30 | Can’t tell | Can’t tell | / | Thought reframing – Coping | Group Activity – Sharing | / |
| Hyer et al.,2009 | Goal Oriented Therapy + Scheduling Pleasant Activities (GIST) | 13 | Care as usual | 12 | Yes | 14 | America | GDS-15 | Yes | Can’t tell | Structure | Goal setting – Learning – Problem solving | Group activity – Individual Contact – Sharing | Activating- Meaningful |
| Konnert et al.,2009 | Cognitive Behavioral Therapy + Scheduling Pleasant Activities | 20 | Care as usual | 23 | Yes | 6 | America | GDS-30 | No | Can’t tell | Structure | Thought reframing – Coping – Learning | Group Activity – Sharing | Activating |
| Lichtenberg et al.,2005 | Scheduling Pleasant Activities | 9 | Care as usual | 11 | No | 12 | America | GDS-15 | Yes | Can’t tell | Breathing exercises, Structure | Imagery – Learning | Individual Contact | Activating |
| Luo et al.,2020 | Scheduling Pleasant Activities (PMAL) | 32 | Care as usual | 30 | Yes | 12 | Asia | GDS-15 | Yes | No | Structure | Goal setting – Problem-solving | Group Activity | Activating |
| Moghadam et al.,2018 | Cognitive Existential Group Therapy (cognitive re-evaluation technique + improving coping skills + existential strategies + supportive instrumental strategies) | 15 | Care as usual | 15 | No | 10 | Asia | GDS-30 | Can’t tell | Can’t tell | / | Thought reframing – Coping | Group Activity | Giving Support |
| Parola et al.,2016 | Cognitive Stimulation Therapy | 49 | Care as usual | 51 | No | 7 | Europe | GDS-15 | Yes | Yes | Small motor skills | Thought stimulation, Memories, Sensory stimulation | Group Activity – Sharing | Encouraging |
| Yang et al.,2016 | Cognitive Stimulation Therapy | 24 | Reminiscence therapy | 40 | No | 10 | Asia | CSDD | Yes | Can’t tell | / | Thought stimulation | Group Activity – Sharing | Encouraging |
| Chiang et al.,2019 | Cognitive Behavioral Therapy (3L-Mind-Training programme: Question Persuade Refer – QPR; change thinking patterns) | 64 | Waiting List | 62 | Yes | 6 | Asia | GDS-15 | Yes | Yes | / | Drawing, Thought reframing – Learning | Group Activity – Sharing | Giving Support, Positivity |
| Davison et al.,2017 | Acceptance and Commitment Therapy | 22 | Waiting List | 19 | Yes | 8 | Australia-New Sealand | GDS-15 | Yes | Can’t tell | Mindfulness – Breathing exercise | Thought stimulation – Imagery – Coping – Goal setting – Problem solving | Individual Contact | Acceptance – encouraging |
| Dozeman et al.,2011 | Scheduling Pleasant Activities | 67 | Care as usual | 62 | No | 12 | Europe | CES-D | Yes | Can’t tell | Structure | Learning | Group Activity | Activating |
| Lin et al.,2019 | Creative Expression (CE – TimeSlips) | 43 | Psychosocial (playing cognitive stimulating games, singing songs or group discussions) | 48 | No | 6 | Asia | CSDD | Can’t tell | No | / | Creativity, Imagery | Group Activity – Sharing, Nature | Encouraging |
| McCurren et al.,1999 | Acceptance and Commitment Therapy | 34 | Care as usual | 27 | Yes | 24 | America | GDS-30 | Yes | Can’t tell | / | Thought reframing – Thought stimulation, Memories – Accomplishments | Individual Contact (volunteer) | Encouraging – Positivity – Acceptance |
| Tsai et al.,2008 | Self-worth Therapy | 31 | Psychosocial (friendly visit) | 32 | No | 4 | Asia | GDS-30 | Yes | Yes | Health education | Coping – Goal setting – Learning, Memories – Accomplishments | Individual Contact | / |
| Wang et al.,2011 | Gerotranscendence Therapy | 35 | Psychosocial (general chatting) | 41 | Yes | 8 | Asia | GDS-15 | Yes | Can’t tell | / | Thought stimulation – Learning, Memories | Group Activity – Sharing | Positivity |
| Wilson et al.,2010 | Hope intervention | 6 | Psychosocial (friendly visit) | 9 | No | 4 | America | GDS-15 | No | Can’t tell | / | Goal setting, Memories | Individual Contact – Sharing | Hope |
| Elsegood et al.,2012 | Mindfulness based Therapy (Guided imagery) | 15 | Care as usual | 15 | No | 2 | Asia | Taiwanese GDS | Yes | Can’t tell | Relaxation | Imagery, Sensory stimulation | Group Activity | Encouraging |
|  |  |  |  |  |  |  |  |  |  |  |  |  |  |  |
| Author(s) | IG | N° IG | CG | N° CG | Sign* | Trial duration* | Continent | Instrument Depression | (Mild) cognitive impairment | Dependent | Body | Mind | Bonding | Positivity |
| Exercise Interventions (N=20) | | | | | | | | | | | | | | |
| Cancela et al.,2016 | Daily cycling sessions | 73 | Care as usual | 116 | No | 60 | Europe | CSDD | Yes | No | Strength | / | Individual or in pair | / |
| Celko et al.,2014 | Dance intervention (EXDASE) | 79 | Waiting List | 83 | Yes | 12 | Europe | GDS-15 | Yes | Yes | Dance/ROM, Breathing exercises | Music listening, Memories | Group Activity | Encouraging |
| Chen et al.,2017 | Band resistance exercise | 65 | Care as usual | 62 | Yes | 24 | Asia | CSDD | Yes | Yes | Aerobic/ Strength/ ROM | / | Group Activity | / |
| Chen et al.,2015 | Wheelchair-bound senior elastic band (WSEB) exercise program | 59 | Care as usual | 55 | Yes | 24 | Asia | Taiwanese GDS | No | Yes | Aerobic/ Strength | / | Group Activity | / |
| Chen et al.,2021 | Otago exercise | 29 | Cognitive intervention (health education) | 29 | Yes | 12 | Asia | GDS-15 | Yes | Can’t tell | Strength/ Balance, Health education | Learning | Group Activity | / |
| Cheng et al.,2011 | Seated Tai Chi | 12 | Psychosocial (playing cognitive stimulating games) | 12 | No | 12 | Asia | GDS-15 | Yes | No | ROM/Balance/Coordination/Tai Chi, Breathing exercises | / | Group Activity | / |
| Hsu et al.,2016 | Tai Chi | 30 | Care as usual | 30 | Yes | 26 | Asia | GDS-15 | No | Yes | TaiChi, Breathing exercises | / | Group Activity | / |
| Chin et al.,2004 | Strength training program | 41 | All round functional training | 48 | No | 24 | Europe | GDS-30 | Can’t tell | Yes | Strength |  | Group Activity |  |
| Conradson et al.,2010 | High-intensity functional weight-bearing exercise programme | 75 | Psychosocial (activities performed while sitting, for example, watching films, singing, reading and conversation) | 90 | No | 12 | Europe | GDS-15 | Yes | Yes | Strength/ Aerobics/ Balance/ADL-training | / | Group Activity | Activating – Encouraging |
| Cordes et al.,2021 | Multicomponential exercise program | 16 | Waiting List | 15 | Yes | 16 | Europe | CES-D | Yes | Yes | ROM/Balance/Strength/ Small motor skills /ADL training/ Aerobic, Relaxation | Thought stimulation, Music | Group Activity | / |
| Eggermont et al.,2009 | Small motor skills (Finger Movements) | 23 | Psychosocial (discussion Group) | 24 | No | 6 | Europe | GDS-30 | Can’t tell | Can’t tell | Small motor skills | / | Group Activity | / |
| Fakhari et al.,2017 | Tai Chi | 27 | Care as usual | 29 | Yes | 12 | Asia | BDI-II | Yes | Yes | TaiChi, Breathing exercise | / | Group Activity | / |
| Krishnamurthy et al.,2007 | Yoga | 18 | Aryuveda | 12 | Yes | 24 | Asia | GDS-15 | Can’t tell | Yes | Yoga, Breathing exercises | Music singing | Group Activity | / |
| Lee et al.,2020 | Qigong | 14 | Cognitive training (cognitive activities involving upper arm mobilization) | 16 | Yes | 12 | Asia | PHQ | Yes | Yes | Yoga, Breathing exercises | / | Individual Contact | / |
| Lok et al.,2017 | Aerobic (walking + rhythmic exercises) | 40 | Care as usual | 40 | Yes | 10 | Europe | BDI-II | Yes | Yes | Aerobic | / | Group Activity | / |
| Tapps et al.,2013 | Resistance Based Physical Activity | 11 | Care as usual | 20 | Yes | 12 | America | BDI-II | Yes | Can’t tell | Strength | / | Group Activity |  |
| Todri et al.,2019 | Global postural reeducation | 45 | Care as usual | 45 | Yes | 24 | Europe | GDS-15 | Yes | Yes | ROM, Breathing exercises | / | Individual Contact | / |
| Tseng et al.,2006 | Range of motion movements | 21 | Range of motion movements | 21 | Yes | 4 | Asia | GDS-15 | No | Can’t tell | ROM | / | Group Activity | / |
| Underwood et al.,2013 | Progressive aerobic and resistance training activities | 224 | Tailored Activities (staff education) | 260 | No | 48 | Europe | GDS-15 | No | Can’t tell | Aerobic/ Strength | / | Group Activity | Activating – Encouraging |
| Williams et al.,2008 | Strength, balance and flexibility exercises | 16 | Psychosocial (social conversation) | 12 | No | 16 | America | CSDD | No | Can’t tell | Strength/ Balance/ROM | / | Group Activity | / |
|  |  |  |  |  |  |  |  |  |  |  |  |  |  |  |
| Author(s) | IG | N° IG | CG | N° CG | Sign.* | Trial duration* | Continent | Instrument Depression | (Mild) cognitive impairment | Dependent | Body | Mind | Bonding | Positivity |
| Green Care (N=11) | | | | | | | | | | | | | | |
| Chu et al.,2019 | Horticultural therapy | 75 | Care as usual | 75 | Yes | 8 | Asia | GDS-15 | No | No | Small motor skills, Touch, Relaxation | Learning, Memories, Sensory stimulation through smell | Group Activity – Sharing, Nature | / |
| Colombo et al.,2005 | Animal Assisted Intervention (taking care of a canary) | 48 | Horticultural (plant) | 43 | Yes | 12 | Europe | BSI | No | No | / | / | Animals | Caretaking |
| Friedman et al.,2015 | Animal Assisted Intervention (Pet Assisted Living Intervention with dog) | 19 | Reminiscence (Life Review) | 18 | No | 12 | America | CSDD | Can’t tell | Can’t tell | Small Motor skills/ROM, Caress an animal, Relaxation | Thought stimulation | Group Activity, Animals, Caretaking | Petting – Caretaking |
| Le roux et al.,2009 | Animal Assisted Activity (Pets as Therapy) | 7 | Care as usual | 8 | Yes | 6 | Africa | BDI-II | Can’t tell | Can’t tell | Small motor skills, Caress an animal | / | Individual Contact, Animals | Petting |
| Olsen et al.,2016 | Animal-Assisted Activity (Dog assisted) | 25 | Care as usual | 26 | No | 12 | Europe | CSDD | Yes | Yes | Small motor skills/ROM, Caress an animal, Relaxation | / | Group Activity, Animals, Caretaking | Petting – Caretaking |
| Parra et al.,2021 | Animal Assisted Intervention | 171 | Care as usual | 163 | Yes | 32 | Europe | CSDD | Yes | Yes | Small motor skills/Upper body, Caress an animal, Relaxation | Thought stimulation, Memories | Group Activity – Sharing, Animals | Petting, Express positive emotions |
| Sollami et al.,2017 | Animal Assisted Intervention | 14 | Care as usual | 14 | Yes | 8 | Europe | GDS-15 | No | Can’t tell | Small motor skills,Caress an animal | Learning | Group Activity, Animals | Petting – Caretaking |
| Thodberg et al.,2016 | Animal Assisted Activity (person visiting with dog) | 35 | Pet-Robot (PARO-visit) | 35 | No | 6 | Europe | GDS-15 | Yes | Can’t tell | Caress an animal, Relaxation |  | Individual Contact, Animals | / |
| Ambrosi et al.,2019 | Animal Assisted Intervention (Dog Assisted Therapy) | 17 | Care as usual | 12 | Yes | 10 | Europe | GDS-15 | Yes | Can’t tell | Small motor skills, Caress an animal | / | Individual Contact, Animals | Petting |
| Travers et al.,2013 | Animal Assisted Intervention (Dog Assisted Intervention) | 27 | Psychosocial | 28 | No | 11 | Australia-New Sealand | GDS-15 | Can’t tell | Can’t tell | Caress an animal | / | Group Activity – Sharing, Animals | Petting – Caretaking |
| Lutwack-Bloom et al.,2008 | Animal Assisted Activity (Dog Visits) | 42 | Psychosocial (Friendly visit) | 26 | No | 24 | Unknown | GDS-15 | Yes | Can’t tell | Caress an animal | / | Group Activity, Animals | / |
|  |  |  |  |  |  |  |  |  |  |  |  |  |  |  |
| Author(s) | IG | N° IG | CG | N° CG | Sign.* | Trial duration* | Continent | Instrument Depression | (Mild) cognitive impairment | Dependent | Body | Mind | Bonding | Positivity |
| Neurobiological Interventions (N=16) | | | | | | | | | | | | | | |
| Badrasawi et al.,2013 | Talbinah food | 30 | Care as usual | 30 | Yes | 7 | Asia | GDS-12 | No | Can’t tell | Nutrition | / | / | / |
| Bergh et al.,2012 | SSRI continuing | 46 | Placebo neuro (SSRI discontinuing) | 31 | No | 25 | Europe | CSDD | Yes | Can’t tell | / | / | / | / |
| Veleva et al.,2020 | Ultra Violet Light | 25 | Neurobiological (Vitamin D supplementation) | 27 | No | 24 | Europe | CSDD | Yes | Yes | / | Sensory stimulation | / | / |
| Dowling et al.,2007 | Light Therapy (morning bright light exposure; >2,500lux) | 29 | Neurobiological (Afternoon bright light exposure; >2,500lux in gaze direction) | 24 | Yes | 10 | America | NPI-NH | Yes | Can’t tell | / | Sensory stimulation | / | / |
| Erdal et al.,2018 | Paracetamol or buprenorphine | 44 | Placebo Neuro | 49 | No | 13 | Europe | CSDD | Yes | Can’t tell | / | / | / | / |
| Hashimoto et al.,2017 | Omega 3 fat acid (1720 mg) | 43 | Placebo neuro (Omega 3 fat acid; 53 mg) | 32 | No | 48 | Asia | Zung Self-Rating Depression Scale | Can’t tell | Yes | Food supplements | / | / | / |
| Kennedy et al.,2000 | Sertraline | 17 | Placebo neuro | 14 | No | 8 | Unknown | CSDD | Yes | Can’t tell | / | / | / | / |
| Kolberg et al.,2021 | Light Therapy (779 lux) | 27 | Placebo neuro (124 lux) | 24 | Yes | 24 | Europe | CSDD | Yes | Yes | / | Sensory stimulation |  |  |
| Luijpen et al.,2018 | Transcutaneous Electrical Nerve Stimulation | 17 | Placebo neuro (Sham stimulation) | 17 | Yes | 6 | Unknown | GDS-30 | Yes | No | / | / | / | / |
| Onega et al.,2018 | Light Therapy (10.000 lux) | 30 | Placebo neuro (Placebo exposure to light) | 30 | Yes | 8 | America | CSDD | No | Can’t tell | / | Sensory stimulation |  |  |
| Rondonelli et al.,2021 | Fish oil | 22 | Placebo neuro (Paraffin oil) | 24 | Yes | 8 | Europe | GDS-30 | Yes | Can’t tell | Nutrition | / | / | / |
| Royer et al.,2012 | Lighttherapy (400 lux) | 15 | Placebo neuro (Light – 75 lux) | 13 | No | 4 | America | GDS-15 | Yes | Yes | / | / | / | / |
| Stange et al.,2015 | Oral Nutritional Supplements (2X Fortimel Compact, 12 g protein and 300 kcal per bottle) | 22 | Care as usual | 20 | No | 12 | Europe | GDS-15 | Yes | Yes | Nutrition | / | / | / |
| Oslin et al.,2003 | Sertraline | 20 | Neurobiological (Venlafaxine) | 12 | No | 10 | America | GDS-30 | Yes | Yes | / | / | / | / |
| van Dongen et al.,2000 | Ginkgo Biloba (240 mg) | 79 | Placebo neuro (no Ginkgo supplement) | 44 | No | 24 | Europe | GDS-15 | Can’t tell | Yes | Food supplements | / | / | / |
| Scilley et al.,2007 | Refractive error correction | 78 | Waiting List | 64 | Yes | 8 | America | GDS-15 | Can’t tell | Can’t tell | / | Sensory stimulation (Visual) | / | / |
| Ulfvarson et al.,2003 | SSRI Discontinuing | 25 | Neurobiological (Continuing SSRI) | 27 | No | 24 | Europe | MADRS | No | Can’t tell | / | / | / | / |
|  |  |  |  |  |  |  |  |  |  |  |  |  |  |  |
| Author(s) | IG | N° IG | CG | N° CG | Sign.* | Trial duration* | Continent | Instrument Depression | (Mild) cognitive impairment | Dependent | Body | Mind | Bonding | Positivity |
| Pet-Robots (N=4) | | | | | | | | | | | | | | |
| Joranson et al.,2015 | PARO group activity | 27 | Care as usual | 26 | No | 12 | Europe | CSDD | Yes | No | Touch | / | Group Activity | Petting, Encouraging |
| Moyle et al.,2013 | PARO group activity | 18 | Psychosocial (Reading activities) | 18 | No | 5 | Australia-New Sealand | GDS-15 | Yes | Yes | Touch | Memories, Sensory stimulation | Group Activity – Sharing | Encouraging |
| Robinson et al.,2013 | PARO group activity | 17 | Psychosocial (City Bus Trips or alternative games) | 17 | No | 12 | Australia-New Sealand | GDS-15 | Yes | Can’t tell | Touch | Thought stimulation | Group Activity – Sharing | / |
| Petersen et al.,2017 | PARO group activity | 35 | Care as usual | 26 | Yes | 12 | Unknown | CSDD | No | Can’t tell | / | / | Group Activity | Encouraging |
|  | | | | | | | | | | | | | | |
| Author(s) | IG | N° IG | CG | N° CG | Sign.* | Trial duration* | Continent | Instrument Depression | (Mild) cognitive impairment | Dependent | Body | Mind | Bonding | Positivity |
| Psychosocial Interventions (N=8) | | | | | | | | | | | | | | |
| Buettner et al.,2002 | Bike program | 35 | Care as usual | 35 | Yes | 12 | America | GDS-15 | Yes | Can’t tell | / | / | Individual Contact, Nature | Activating |
| Carroll et al.,1998 | Laughter Therapy | 31 | Care as usual | 30 | Yes | 5 | Europe | HADS | Can’t tell | Can’t tell | Dance | Music listening – Singing | Group Activity | Laughing – Encouraging |
| Chiu et al.,2019 | ICT-communication | 19 | Cognitive intervention (ICT- entertainment) | 18 | Yes | 12 | Asia | CES-D | No | Yes | / | Creativity, Learning, Music listening | Group Activity – Sharing | / |
| Hsu et al.,2019 | Social supportive activities programs (social activities, e.g., playing games, learn to work on Ipad) | 35 | Care as usual | 33 | Yes | 10 | Asia | GDS-15 | Yes | Can’t tell | / | Learning | Group Activity – Sharing | Giving Support |
| Low et al.,2012 | LaughterBoss training | 174 | Care as usual | 197 | No | 13 | Australia-New Sealand | CSDD | Can’t tell | Can’t tell | / | Memories | Group Activity | Positivity |
| Siregar et al.,2019 | Laughter therapy | 21 | Care as usual | 21 | Yes | 4 | Asia | GDS-15 | Can’t tell | Can’t tell | / | / | Group Activity | Positivity |
| Tsai et al.,2011 | Videoconference Program | 40 | Care as usual | 50 | No | 48 | Asia | GDS-30 | No | Yes | / | Learning | Individual Contact (Family) | / |
| Tse et al.,2017 | Play activities (social pleasant group activities) | 29 | Placebo (Reading activities; no interaction) | 24 | No | 8 | Asia | GDS-15 | Yes | Yes | Exercise | Thinking (gaming) | Group Activity | Positivity |
|  |  |  |  |  |  |  |  |  |  |  |  |  |  |  |
| Author(s) | IG | N° IG | CG | N° CG | Sign.* | Trial duration* | Continent | Instrument Depression | (Mild) cognitive impairment | Dependent | Body | Mind | Bonding | Positivity |
| Reminiscence (N=16) | | | | | | | | | | | | | | |
| Bailey et al.,2017 | Qar intervention | 26 | Psychosocial (Pleasant group activities) | 25 | Yes | 6 | America | GDS-30 | Yes | Yes | / | Creating audiotape, Thought stimulation, Memories | Group Activity – Sharing | / |
| Chiang et al.,2010 | Series of Life Review interventions | 45 | Waiting List | 47 | No | 8 | Asia | CES-D | Yes | Can’t tell | / | Thought reframing – Goal setting, Memories – Accomplishments | Group Activity – Sharing | Positivity |
| Cook, E. A.,1991 | Positive reminiscence group therapy | 14 | Psychosocial (Talking about current events) | 13 | No | 16 | America | GDS-30 | No | Yes | / | Music listening, Memories | Group Activity – Sharing | Positivity |
| Elias et al.,2020 | Spiritual reminiscence | 18 | Psychosocial (Pleasant group activities) | 16 | No | 6 | Asia | GDS-14 (Malay) | No | Can’t tell | / | Memories | Group Activity – Sharing | Hope – Meaningful |
| Goldwasser et al.,1987 | Positive reminiscence group therapy | 29 | Psychosocial (Talking about current events) | 29 | No | 5 | America | BDI-II | Yes | No | / | Thought reframing, Music listening, Memories, Sensory stimulation through food, smell | Group Activity – Sharing | Positivity |
| Hsieh et al.,2010 | Life Review | 29 | Care as usual | 32 | Yes | 12 | Asia | GDS-15 | Yes | Can’t tell | / | Memories | Group Activity – Sharing | Encouraging – Positivity |
| Hsu et al.,2009 | Group structured reminiscence | 21 | Care as usual | 24 | Yes | 8 | Asia | GDS-15 | Yes | Can’t tell | / | Music listening, Memories, Sensory stimulation through food | Group Activity – Sharing | / |
| Lan et al.,2019 | Structured Life Review | 31 | Care as usual | 31 | Yes | 6 | Asia | GDS-15 | No | No | / | Thought reframing, Memories | Group Activity – Sharing | Positivity – Acceptance |
| Lok et al.,2004 | Life Review | 30 | Care as usual | 30 | Yes | 8 | Europe | CSDD | Yes | Can’t tell | / | Music listening, Memories – Accomplishments, Sensory stimulation through food | Group Activity – Sharing | positivity |
| Lopes et al.,2016 | Unstructured Life Review | 20 | Care as usual | 20 | Yes | 5 | Europe | GDS-5 | Yes | Can’t tell | / | Thought stimulation, Memories | Individual Contact – Sharing | Encouraging – Positivity |
| Soniya, G.,2013 | Positive reminiscence group therapy | 30 | Care as usual | 30 | Yes | 1 | Asia | GDS-30 | Can’t tell | Can’t tell | / | Memories – Accomplishments | Group Activity – Sharing | pleasurable |
| Van Bogaert et al.,2016 | Structured individual reminiscence intervention (based on the SolCos model) | 29 | Care as usual | 31 | Yes | 9 | Europe | CSDD | Yes | Can’t tell | / | Thought reframing, Memories, Sensory stimulation | Individual Contact – Sharing | Giving Support, Meaningful |
| Wang et al.,2007 | Group structured reminiscence | 51 | Care as usual | 51 | No | 8 | Asia | GDS-15 | Yes | Can’t tell | / | Music listening, Memories – Accomplishments, Sensory stimulation through food | Group Activity – Sharing | / |
| Westerhof et al.,2018 | Individual Precious memories Therapy | 42 | Psychosocial (Friendly visit) | 39 | No | 8 | Europe | GDS-8 | Can’t tell | Can’t tell | / | Memories | Individual Contact – Sharing | Positivity |
| Meléndez-Moral et al.,2013 | Group structured reminiscence | 17 | Care as usual | 17 | Yes | 7 | Europe | GDS-8 | No | Can’t tell | / | Memories | Group Activity | / |
| Hamzehzadeh et al.,2018 | Integrative reminiscence and instrumental group reminiscence | 10 | Care as usual | 11 | Yes | 4 | Asia | GDS-15 | Can’t tell | Can’t tell | / | Creativity, Thought reframing – Goal setting – Problem solving, Memories | Group Activity – Sharing | Pleasant memories – Meaningful |
|  |  |  |  |  |  |  |  |  |  |  |  |  |  |  |
| Author(s) | IG | N° IG | CG | N° CG | Sign.* | Trial duration* | Continent | Instrument Depression | (Mild) cognitive impairment | Dependent | Body | Mind | Bonding | Positivity |
| Sensory Stimulation (N=19) | | | | | | | | | | | | | | |
| Sanchez et al.,2016 | Multisensory stimulation (Snoezelen) | 9 | Sensory Stimulation (Preferred Music Listening) | 10 | No | 16 | Europe | CSDD | Yes | Can’t tell | Touch | Sensory Stimulation - Aromatherapy | / | Encouraging |
| Aravich et al.,2021 | Multisensory (Medium : Computer) | 4 | Care as usual | 6 | Yes | 12 | America | GDS-15 | Yes | Can’t tell | Exercise, Touch | Learning, Music listening | / | Laughing - Humour |
| Aravich et al.,2021 | Multisensory (Medium : Computer) | 8 | Care as usual | 10 | Yes | 12 | America | GDS-15 | Yes | Can’t tell | Exercise, Touch | Learning, Music listening | / | Laughing - Humour |
| Bae et al.,2020 | Environmental (Olfactory stimulation with lavender) | 21 | Placebo group (non scented oil) | 21 | No | 2 | America | GDS-15 | Can’t tell | Can’t tell | / | Sensory stimulation - Aromatherapy | Nature | / |
| Ching-Teng, et al.,2019 | Art therapy (based on expressive therapy continuum and media dimension variables) | 29 | Care as usual | 26 | Yes | 12 | Asia | GDS-15 | Yes | Yes | Small motor skills | Creativity, Thought stimulation - Imagery, Memories - Accomplishments, Sensory stimulation | Group Activity - Sharing - Family | / |
| Cooke et al.,2010 | Live group music programme delivered by two musicians | 24 | Psychosocial (Reading group) | 23 | No | 8 | Australia-New Sealand | GDS-15 | Yes | Can’t tell | Dance | Music listening - Singing - Playing instruments | Group Activity | Encouraging |
| Costa et al.,2018 | Preferred Music Listening | 55 | Care as usual | 58 | Yes | 3 | Europe | PHQ-9 | Can’t tell | Can’t tell | / | Music listening | / | / |
| Davison et al.,2016 | Multisensory (Medium : Memory Box) | 11 | Psychosocial (Discussion group) | 11 | No | 4 | Australia-New Sealand | CSDD | No | Can’t tell | / | Music listening, Memories | / | / |
| Ugur et al.,2016 | Music Listening group activity | 32 | Care as usual | 32 | Yes | 8 | Asia | GDS-15 | Yes | Can’t tell | / | Imagery, Music listening, Memories | Group Activity | Happy memories |
| Alka et al.,2021 | Multisensory Stimulation Program (SONAS) | 48 | Psychosocial (Reading group) | 32 | No | 24 | Europe | CSDD | No | Can’t tell | Exercise, Massage | Thought stimulation, Music Listening - Singing - Playing instruments, Sensory Stimulation (Taste and Touch) | Group Activity - Sharing | / |
| Hutson et al.,2014 | Multisensory Stimulation Program (SONAS) | 20 | Care as usual | 16 | No | 7 | Europe | CSDD | Yes | Can’t tell | Exercise, Massage | Thought stimulation, Music Listening - Singing - Playing instruments, Sensory Stimulation (Taste and Touch) | Group Activity - Sharing | / |
| Hyun-Sil Kim et al.,2021 | Playing Korean Trot Music and Rythmic Exercises | 20 | Care as usual | 20 | Yes | 12 | Asia | GDS-15 | No | Can’t tell | Dance/ROM | Music listening - Singing, Memories | Group Activity | / |
| Moghaddasifar et al.,2018 | Multisensory stimulation (Tactile, Auditory, and visual stimulation) | 14 | Care as usual | 14 | Yes | 4 | Asia | BDI-II | Yes | Can’t tell | Massage | Music listening, Sensory stimulation | Individual Contact, Nature | / |
| Pérez-Ros et al.,2019 | Preferred Music Listening | 47 | Care as usual | 72 | Yes | 8 | Europe | GDS-15 | Yes | Can’t tell | Dance | Music listening - Singing | Group Activity | / |
| Rodriguez-Mansilla et al.,2015 | Ear Accupressure | 40 | Sensory stimulation (Massage Therapy) | 35 | No | 12 | Europe | CSDD | Yes | Can’t tell | Touch, Relaxation | Sensory stimulation | Individual Contact | / |
| Teng et al.,2021 | Auricular Acupressure | 20 | Placebo Intervention (blank patches) | 19 | Yes | 2 | Asia | GDS-15 | Yes | Yes | Touch | Sensory stimulation | / | / |
| Werner et al.,2017 | Interactive Music Therapy (based on holistic approach of Muthesius) | 62 | Sensory Stimulation (recreational singing) | 53 | Yes | 12 | Europe | MADRS | Yes | Can’t tell | ROM/ Playing instruments | Improvising,Music Listening - Singing - Playing instruments,Memories | Group Activity | / |
| Biasutti,2021 | Rhythm-music and music improvisation exercises | 20 | Exercise (gymnastic activities) | 25 | No | 6 | Europe | GDS-15 | Yes | Can’t tell | Dance | Creativity, Thinking - Learning, Music listening - Singing | Group Activity - Sharing | / |
| Baker et al.,2022 | Group Music Therapy | 77 | Sensory Stimulation (Recreational Choir Singing) | 82 | Yes | 24 | Australia-New Sealand | MADRS | No | Can’t tell | Exercise | Improvising,Music listening - Singing, Memories | Group Activity | / |
| Costa et al.,2018 | Preferred Music Listening | 55 | Care as usual | 58 | Yes | 3 | Europe | PHQ-9 | No | Can’t tell | / | Music listening | / | / |
| Author(s) | IG | N° IG | CG | N° CG | Sign* | Trial duration* | Continent | Instrument Depression | (Mild) cognitive impairment | Dependent | Body | Mind | Bonding | Positivity |
| Tailored interventions (N=6) | | | | | | | | | | | | | | |
| Chapman et al.,2007 | Tailored careplanning ((1) medical, (2) meaningful activities, (3) psychological, and (4) behavioral concerns) | 57 | Waiting List | 61 | No | 8 | America | CSDD | Yes | Can’t tell | / | / | Group Activity | Meaningful – Support |
| Llewellyn-Jones et al.,1999 | Shared Care Intervention (Combination Staff education, health education and health promoting activities) | 86 | Care as usual | 83 | Yes | 38 | Australia-New Sealand | GDS-30 | No | Can’t tell | Health education | Thought reframing - Learning | Group Activity - Sharing | Activating |
| Proctor et al.,1999 | Staff training and of psychosocial management of residents’ behavioural problems (based on goals planning activities) | 54 | Care as usual | 51 | Yes | 24 | Europe | AGECAT depression | Can’t tell | No | / | Thinking | Group Activity | Activating |
| Resnick et al.,2010 | FFC-AL-EIT (Staff Training, care service plans, mentoring, and motivating) | 321 | Care as usual | 229 | Yes | 48 | America | CSDD | No | Can’t tell | Exercise | Learning | Group Activity | Activating - Encouraging |
| Alidoost et al.,2021 | An individualized four‑session care plan sessions | 29 | Care as usual | 30 | Yes | 10 | Asia | GDS-15 | No | Can’t tell | Health education | Thought reframing – Coping - Learning | Group activity - Individual Contact | Activating - Positivity |
| McSweeney et al.,2012 | Consultation regarding best-practice management of depression to facility staff and developed a tailored, psychosocial care plan | 17 | Care as usual | 22 | Yes | 15 | Australia-New Sealand | CSDD | No | Can’t tell | Health education - Structure | Music listening, Memories, Sensory stimulation | Individual Contact | Activating |

*Note. ^a^A study is significant when the between group differences postintervention were significant; ^b^Trial duration = in weeks; IG = Intervention Group; CG = Control Group; N° IG/CG = number of participants in the intervention/control group; CSDD = Cornell Scale for Depression in Dementia; GDS = Geriatric Depression Scale; MADRS = Montgomery Asberg Depression Rating Scale; HADS =* *Hospital Anxiety and Depression Scale; CES-D = Center for Epidemiologic Studies Depression scale; BDI = Beck Depression Inventory; AGECAT = Automated Geriatric Examination for Computer Assisted Taxonomy*
